# Unravelling cucumber resistance to several viruses via genome-wide association studies highlighted resistance hotspots and new QTLs

**DOI:** 10.1093/hr/uhac184

**Published:** 2022-08-25

**Authors:** Severine Monnot, Melissa Cantet, Tristan Mary-Huard, Laurence Moreau, Rebecca Lowdon, Maurine Van Haesendonck, Agnès Ricard, Nathalie Boissot

**Affiliations:** INRAE, Génétique et Amélioration des Fruits et Légumes, 84143, Montfavet, France; Bayer Crop Science, 13670, Saint-Andiol, France; Bayer Crop Science, 13670, Saint-Andiol, France; Université Paris-Saclay, INRAE, CNRS, AgroParisTech, Génétique Quantitative et Evolution - Le Moulon, 91190, Gif-sur-Yvette, France; Université Paris-Saclay, INRAE, CNRS, AgroParisTech, Génétique Quantitative et Evolution - Le Moulon, 91190, Gif-sur-Yvette, France; Université Paris-Saclay, INRAE, AgroParisTech, Mathématiques et Informatique Appliquées-Paris, 75005 Paris, France; Bayer Crop Science, 700 Chesterfield Parkway West, Chesterfield, MO 63017, USA; Bayer Crop Science, 13670, Saint-Andiol, France; INRAE, Génétique et Amélioration des Fruits et Légumes, 84143, Montfavet, France

## Abstract

The mapping and introduction of sustainable resistance to viruses in crops is a major challenge in modern breeding, especially regarding vegetables. We hence assembled a panel of cucumber elite lines and landraces from different horticultural groups for testing with six virus species. We mapped 18 quantitative trait loci (QTL) with a multiloci genome wide association studies (GWAS), some of which have already been described in the literature. We detected two resistance hotspots, one on chromosome 5 for resistance to the cucumber mosaic virus (CMV), cucumber vein yellowing virus (CVYV), cucumber green mottle mosaic virus (CGMMV) and watermelon mosaic virus (WMV), colocalizing with the RDR1 gene, and another on chromosome 6 for resistance to the zucchini yellowing mosaic virus (ZYMV) and papaya ringspot virus (PRSV) close to the putative VPS4 gene location. We observed clear structuring of resistance among horticultural groups due to plant virus coevolution and modern breeding which have impacted linkage disequilibrium (LD) in resistance QTLs. The inclusion of genetic structure in GWAS models enhanced the GWAS accuracy in this study. The dissection of resistance hotspots by local LD and haplotype construction helped gain insight into the panel’s resistance introduction history. ZYMV and CMV resistance were both introduced from different donors in the panel, resulting in multiple resistant haplotypes at same locus for ZYMV, and in multiple resistant QTLs for CMV.

## Introduction

Cucumber is one of the five most cultivated vegetables worldwide. Cucumbers and gherkins were grown on 2.23 million ha of cropland in 2019, representing around 88 million t of harvested produce [5]. The natural adaptation potential of the cucumber species enables its cultivation in diverse environments and farming systems, ranging from glasshouses, with the “Long Dutch” type, to open fields with slicer and Asian types (Pitrat, 2012). Cucumbers can be infected by at least 59 virus species [13] and the globalization trend favors the spread of new virus strains and species. Few quantitative trait loci (QTLs) conferring resistance to viruses have been mapped in segregating cucumber populations (Supplementary Table 1). A QTL for resistance to the zucchini yellowing mosaic virus (ZYMV), papaya ringspot virus (PRSV) and watermelon mosaic virus (WMV) was mapped on chromosome 6 [25, 28, 29], while resistance to the cucumber mosaic virus (CMV) was shown to be either monogenic or polygenic [16]. Silencing the RNA-dependent RNA polymerase 1b (RDR1b) gene, located on chromosome 5, confers cucumber resistance to CMV, cucumber vein yellowing virus (CVYV) and cucumber green mottle mosaic virus (CGMMV) [10, 11]. The eukaryotic initiation factor 4E (EIF4E) gene on chromosome 1 modified by CRISPR-cas9 conferred resistance to CMV [2]. Monogenic resistances have already been mapped and/or introduced in cucumber elite germplasm [16]. However, viruses relatively easily challenge these monogenic resistances while polygenic resistances are expected to be more durable [24].

Genetic studies have so far only considered a tiny portion of the cucumber diversity, while not assessing the overall architecture of cucumber resistance to viruses. Genome-wide association studies (GWAS) conducted in large diversity panels could overcome this limitation, with QTLs mapped by associating genetic variants with specific phenotypic variations. Nevertheless, to date GWAS have seldom been used to map plant-virus resistance due to several difficulties [18]. The latter are mostly related to phenotyping protocols and unbalanced frequencies of resistance alleles from one plant genetic group to another due to plant-virus coevolution.

We addressed all of these aspects by pooling landraces and elite lines into a panel that we tested for its response to three viruses from the potyviridae family (ZYMV, PRSV, WMV and CVYV), one cucumovirus (CMV) and one tobamovirus (CGMMV). We implemented a multilocus GWAS using the reference genome from the “Cornell Chinese Long” (CCL) landrace [12]. We analyzed the main identified QTLs in depth to describe the architecture of resistance to viruses and highlighted different histories of resistance introduction, depending on the virus. This generated crucial information that could be to take full advantage of current cucumber resistance levels.

## Results

 

## The cucumber panel exhibited highly variable distributions of resistance to the six viruses

The panel pooled 226 elite lines from Bayer germplasm collections, 40 landraces from Bayer internal collections and 23 hybrids, including resistance controls (Supplementary Table 2).

The panel was inoculated with six viruses (Table 1) and the plant response was scored in nine ordered classes from 1–3 highly resistant, from 4–7 intermediate and over 7 susceptible. The predicted genetic values per accession were obtained using a Poisson generalized linear model and called Poisson BLUP (PoP). Broad sense heritabilities [3] ranged from 0.93 (WMV) to 0.99 (ZYMV) (Table 1), thereby validating the experimental protocols. Hybrid controls gave the expected phenotypes, except in the WMV and PRSV trials for which intermediate controls gave resistant scores, suggesting that the high resistance levels might have been overestimated for these two viruses in some panel accessions (Supplementary Table 2).

PoP distributions were bimodal, Gaussian, or zero-inflated Poisson (Supplementary Figure 1). PRSV and ZYMV PoPs were closely correlated (0.95), suggesting common genetic control of resistance (Supplementary Figure 1). Only four accessions were resistant to the six viruses, i.e. three landraces (X18CPBLP0004, X18CPBLP0029, X18CPBLP0282) and the elite line X18CPBLP0278 (Supplementary Table 3). The “TMG-1” landrace was highly resistant to all viruses except CGMMV, for which a few symptoms were observed.

## Genetic structure and kinship analyses revealed the different horticultural groups and breeding germplasm

The genotypic datasets are summarized in Table 1, Supplementary Table 4 and Supplementary Figure 2. The genotype matrix (M_NA10_) contained around 1 SNP every 166 bp after application of the different filters (NA < 10%, He<15%, MAF > 0.03%) (Supplementary Table 4). However, we observed a 1.7 Mb gap on chromosome 5 without SNPs. The average LD extent in the panel ranged from 65 kb (chr4) to more than 1 Mb (chr2) (Supplementary Table 4). We observed LD extent disparities between either chromosomes and horticultural groups (Supplementary Figure 2 and Supplementary Table 4). The LD extent was 120 kb shorter in landraces than in elite lines.

**Figure 1:**
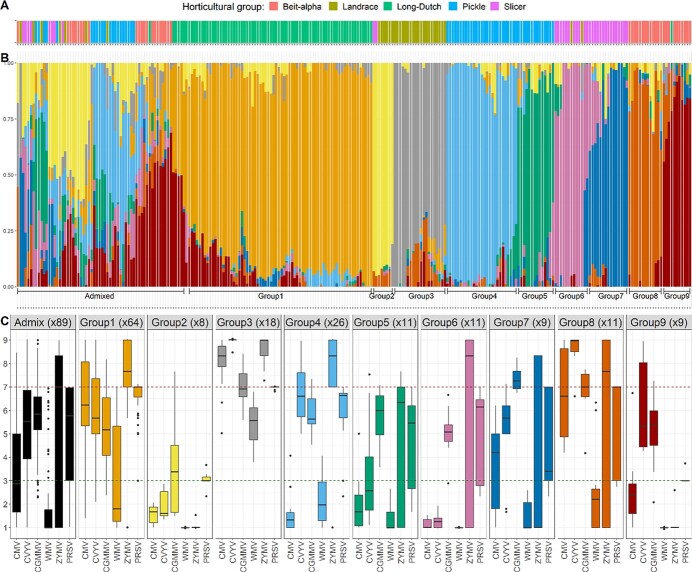
Organization of the 256 cucumber accessions across nine genetic groups **A.** Each color represents the horticultural group (when known) of the accession. **B.** Barplot representing the percentage attribution of 256 cucumber accessions to nine genetic groups. Accessions were rarely attributed 100% to one genetic group and accessions considered as admixed resulted from crosses between breeding programs. Values were extracted with the snmf R package. Genetic groups were consistent with the horticultural groups. **C.** Boxplot representing the phenotypic variance in the diversity panel for each virus. Accessions were assigned to a genetic group when the contribution of this group was above 0.6, but if no group reached this threshold the accession was considered as admixed (in black). The number of accessions per group is indicated in brackets.

The 256 accessions belonged to at least five horticultural groups (Figure 1.A.). Their genetic structure was investigated with the “snmf” algorithm [7]. Five groups matched the horticultural groups (Supplementary Figure 3) while nine groups matched breeding programs (Figure 1). The cross-entropy criterion suggested that more than nine groups provided little additional information (Supplementary Figure 4). For nine groups (Figure 1), landraces were pooled into two genetic groups, i.e. one with accessions from Asia and the other with accessions from Europe and America. The seven other groups included elite lines from different horticultural groups: Long-Dutch (group 1), pickles (groups 4 and 5), slicers (groups 6 and 7) and Beit-alpha (groups 8 and 9) (Figure 1. A.&B.).

A hierarchical clustering of the kinship matrix confirmed the nine-group genetic structure (Supplementary Figure 5). Accessions from genetic group 2, i.e. Asian landraces, had strong kinship coefficients within their group (~3) compared to the rest of the population, thereby highlighting the presence of specific alleles in this group. They also had low kinship coefficients (~ − 1) with some accessions from group 1 (Long-Dutch), thus highlighting their genetic distance with these elite lines.

## Resistant phenotypes were unequally distributed among the different genetic structure groups

**Figure 2:**
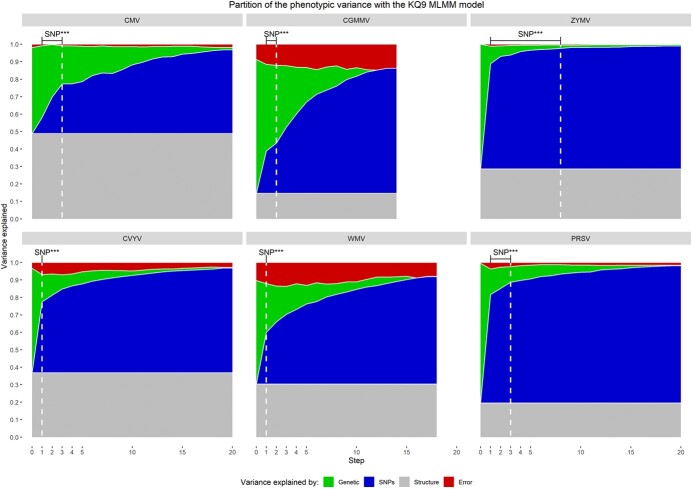
The phenotypic variance distribution among i/ fixed effects: both the genetic structure (grey) and top SNPs from previous MLMM steps (blue) ii/ random effect (genetic, green) and iii/ error (red). The white dashed line indicates significant SNPs using Bonferroni correction. MLMM for CGMMV and WMV respectively stopped at the 14th and 18th steps because no further phenotypic variance explained by the genetic remained after the previous step.

The nine-group genetic structure explained 14% (CGMMV) to 49% (CMV) of the phenotypic variance (PV) depending on the virus (in grey in Figure 2). The GWAS results were interpreted in light of the strong structure depicted here and the distribution of resistant accessions across the different genetic groups (Figure 1.C.).

**Table 1:** Characteristics of the experiment according to the inoculated virus: experimental design and heritability, population composition and genomic variant number filtered for GWAS.

	CMV	CVYV	CGMMV	WMV	ZYMV	PRSV
Phenotyped population						
Elite lines and landraces	261	248	263	261	258	254
F1	16	16	16	16	16	16
F1 commercial	7	7	7	7	7	7
Total	284	271	286	284	281	277
Experimental design						
Location^1^	BHK	BHK	BHK	STA	STA	STA
Structure^2^	1 GH	1 GH	3 GH	1 GH	2 GC	1 GH
S control	Check03	Check03	Check03	Check06	Check07	Check08
IR control	X18CPBLP0041^3^	Check04	Check07 & Check01	Check05		Check02
R control	X18CPBLP0029^3^	X18CPBLP0029^3^	X18CPBLP0282^3^	Check02	Check02	X18CPBLP0029^3^
Plant/accession	15	15	12	15	15	15
Exp. heritability (H^2^)	0.98	0.95	0.93	0.93	0.98	0.99
Phenotyped and genotyped population						
Elite lines and landraces	250	240	245	246	243	241
Number of SNP (M_*NA*10_)	1,271,848	1,271,848	1,271,848	1,271,848	1,271,848	1,271,848
Number of SNP (M_*NA*00_)	376,151	422,431	383,943	387,164	420,504	418,497

^1^BHK: Bergschenhoek; STA: Saint-Andiol

^2^GH: Glasshouse; GC: Growth chamber

^3^X18CPBLP0029, X18CPBLP0041 and X18CPBLP0282 are landraces included in the GWAS panel

Group 2 landraces were highly resistant to all viruses but CGMMV, while group 3 landraces were highly susceptible to all viruses. Remarkably, elite-line genetic groups displayed more individuals that were admixed with group 2 landraces than those of group 3 (Figure 1.C), suggesting that group 2 landraces were used as resistance donors. Group 2 resistant alleles might therefore be frequent enough in the panel to reach the MAF threshold.

Elite lines from genetic groups 4, 5, 6, and 9 were highly resistant to CMV, while accessions from other groups displayed phenotypic variance. For CVYV, group 6 accessions were highly resistant, while group 8 accessions were highly susceptible. For CGMMV, group 7 and 8 accessions were highly susceptible, and those of the other genetic groups displayed intermediate resistance. For WMV, most accessions were resistant, except those in group 1 and admixed accessions. For ZYMV, group 9 accessions were highly resistant, while group 1, 4 and 5 accessions were susceptible. For PRSV, group 9 accessions were highly resistant, while group 1 and 4 accessions were susceptible. In conclusion, resistant accessions were unequally distributed among genetic groups and intragroup phenotypic variability was low.

## GWAS mapped two clusters of resistance aside isolated QTLs

 

## Detection of resistance hotspots and isolated resistant QTLs by MLMM

An iterative GWAS procedure (MLMM) that introduced the most significant SNPs from previous steps as fixed effect was used [26]. Five models were tested using PoPs as aggregated phenotypes: Q5, Q9, K, KQ5 and KQ9. *P*-values obtained in the MLMM first step via the model that included both the kinship and nine genetic groups (KQ9) generated the curve that best fit the bisector on the QQplot whatever the virus (Supplementary Figure 6). Eighteen QTLs were detected and their intervals and related top SNP information, including physical position, minor allele frequencies (MAF), resistance allele frequencies within each genetic group, are summarized in Supplementary Table 5.

**Figure 3:**
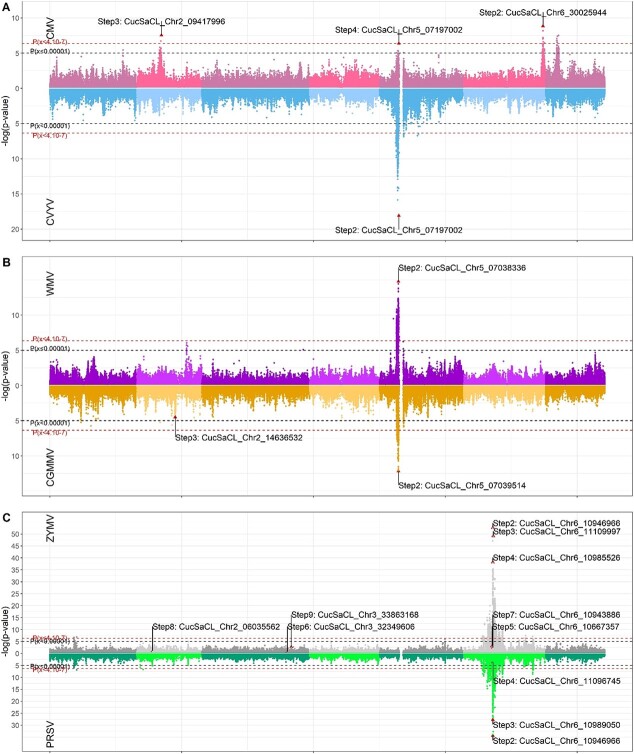
**A** Miami plot consisting of two mirrored Manhattan plots corresponding to two different GWAS. The two significance thresholds are represented by the two dashed lines. These Miami plots were built from *p*-values collected from the first KQ9 MLMM step (without marker correction). The 18 SNPs selected in one of the MLMM steps were represented by a red triangle, their positions as well as the step in which they were selected are labelled. **A.** CMV (pink) and CVYV (blue) shared a common peak and a common top SNP on chromosome 5, CMV had two other isolated QTLs on chromosomes 2 and 6. The peak on chr7 was finally not selected during the MLMM steps as it disappeared as it explained the same phenotypic variance as the peak on chr6 **B.** CGMMV (yellow) and WMV (purple) shared the same peak on chromosome 5 but had different top SNPs. CGMMV presented another QTL on chromosome 2 when tested independently from the top SNP on chromosome 5, even with the lowest threshold. **C.** ZYMV (grey) and PRSV (green) shared two common peaks, i.e. a large one on chromosome 6, with the same top SNPs, and a small one on chromosome 1 that was not selected in further MLMM steps.

Three QTLs were detected for CMV resistance (Figure 3.A. upper graph), with all having a top SNP MAF > 0.4. They explained 7 to 11% of the PV (in blue in Figure 2). The QTL_CmvCH2_ located on chr2 was 1.9 Mb wide, the QTL_CmvCH5 (_1.1 Mb) was mapped just upstream of the SNP gap on chr5 and the QTL_CmvCH6_ located at one end of the chr6 was 1.6 Mb QTL wide.

Only one QTL was detected for CVYV and WMV resistance, i.e. QTL_WmvCH5_ and QTL_CvyvCH5_, both of which were 1.1 Mb wide and located just upstream of the SNP gap on chromosome 5 (Figure 3.A.B. lower graph). Their top SNP explained 40% and 29% of the PV for CVYV and WMV resistance (Figure 2).

Two QTLs were detected for CGMMV resistance, and their top SNPs explained 24 and 5% of the PV (Figure 2). The QTL_CgmmvCH5,_ located upstream of the SNP gap on chr5, was 1.2 Mb wide and QTL_CgmmvCH2_ was only 5.8 kb wide (Figure 3.B. upper graph), while this last QTL barely reached the significance threshold and had an MAF of 0.07.

Eight QTLs were mapped for ZYMV resistance, i.e. five on chr6 that were hardly distinguishable from each other in the Miami plots (Figure 3.C. upper graph), thus questioning whether different QTLs were actually present or if it was simply one very large QTL. In these five QTLs, one top SNPs explained 62% of the PV (Figure 2), with all others having an effect <4%. The three other QTLs were mapped on chr2 and chr3 but only the latter, QTL_ZymvCH3_02_, had an MAF > 0.05 (Supplementary Table 5).

Three overlapping QTLs were mapped for PRSV on chr6 (Figure 3.C. lower graph), with two of them having an MAF of over 0.05 (Supplementary Table 5). Like ZYMV, the first top SNP explained a large part (60%) of the PV, all following top SNPs having an effect <4% (Figure 2) and disparate MAFs (0.34 and 0.04).

QTLs controlling the response to one virus species were either isolated or clustered. Remarkably, most resistance QTLs detected here were colocalized with a resistance QTL to another virus. Hereafter, for these QTLs, we defined resistance hotspots as the colocalization of several QTLs controlling the response to different viruses. Broad-spectrum resistance was a particular case of resistance hotspots where different QTLs shared exactly the same top SNP, suggesting a common resistance gene.

QTLs for resistance to CMV, CVYV, CGMMV and WMV colocalized on chr5 and defined a first resistance hotspot (Table 2). This hotspot on chr5 was 1.1 Mb wide, which was much longer than the chr5 LD extent (0.19 Mb), and it was located near the SNP gap. At this hotspot, resistance QTLs to CMV and CVYV shared the same top SNP, i.e. CucSaCL_Chr5_07197002 (Figure 3.A.), and therefore represented broad-spectrum resistance. QTL_CGMMVCH5_ and QTL_WMVCH5_ top SNPs were located 1.2 kb apart and 160 kb from the QTL_CMV&CVYVCH5_ top SNP. Among the 16 hybrids derived from elite lines of the panel, nine were heterozygous for the hotspot on chr5, thus suggesting an additive effect for CVYV resistance and a dominant effect for WMV resistance in the case of single locus control (Supplementary Figure 7.A.). The allelic interaction for CMV and CGMMV resistance was not resolved due to the presence of other resistance QTLs.

**Table 2:** Summary of the different QTLs mapped by MLMM GWAS

$\includegraphics{\bwartpath uhac184t2}$

The overlapping QTLs detected for ZYMV and PRSV on chr6, respectively pooling five and three QTLs, formed a second resistance hotspot (Table 2) of 3.3 Mb, i.e. wider than the chr6 LD extent (Supplementary Table 4). One top SNP was common for both viruses, CucSaCL_Chr6_1094966 (Figure 3.C) and defined broad-spectrum resistance to ZYMV and PRSV. The top SNPs from the six other QTLs of this cluster, i.e. two for PRSV and four for ZYMV, were different. Seven hybrids were heterozygous for this hotspot, thereby suggesting an additive allelic interaction for ZYMV and PRSV resistance (Supplementary Figure 7.B.)

## Local LD studies and annotation analyses in QTLs revealed potential candidate resistance genes.

**Figure 4:**
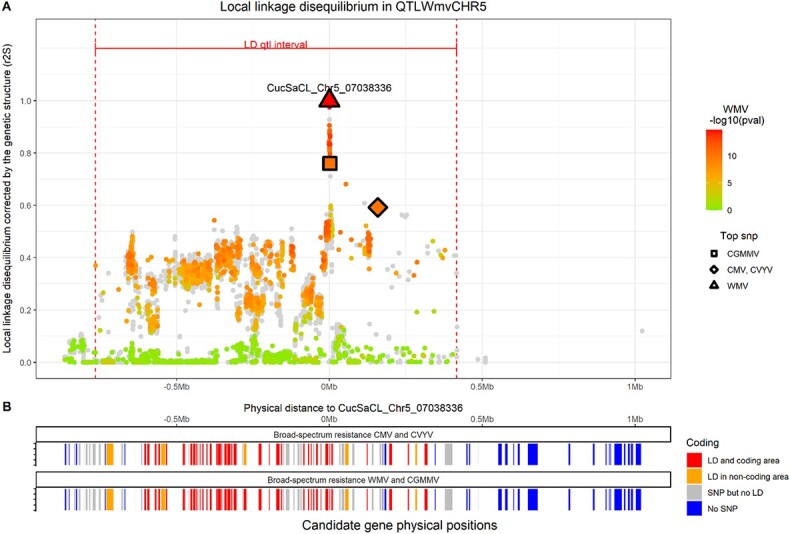
The hotspot on chr 5 **A.** Representation of the LD between the WMV top SNP and all other SNPs at the hotspot on chr5. The X-axis represents the physical distance from the WMV top SNP, while the Y-axis represents the r^2^ corrected by the structure between SNPs and the top SNP. Each dot represents an SNP which is colored according the *p*-value in the first step of MLMM GWAS for WMV. Dots are grey when the SNP was not tested by GWAS due to missing data. The dot shape represents the top SNP of the QTL or top SNPs from other resistance QTLs. The QTL interval is delineated by the red dashed line. **B.** Genes located in the QTL, candidate genes in red (at least one SNP in LD with the top SNP in a coding area), potential candidate genes in orange (at least one SNP in LD with the top SNP in a non-coding area), with other genes in grey or blue. The first line represented these categories for broad spectrum resistance to CMV and CVYV and below for broad-spectrum resistance to CGMMV and WMV.

We used the M_NA10_ genotype matrix to calculate the LD between each top SNP and all physically close SNPs. We considered that an SNP was independent from the top SNP when their r^2^S_Top SNP_ was below 0.2. Otherwise, we retrieved gene annotations from a public database for each genomic area containing the different QTLs (Supplementary Table 6). The number of genes in QTL intervals ranged from 61 (QTL_ZymvCH2_) to 474 (QTL_ZymvCH6_03_ and QTL_PrsvCH6_02_) (Supplementary Table 5). Then we defined four categories of genes: genes with at least one SNP in strong LD with the top SNP being in a coding (red in Figure 4) or non-coding (orange) sequence, genes with at least one SNP not in LD with the top SNP (grey) and genes without SNPs (blue).

In the chr5 hotspot of resistance to four viruses, we analyzed LD from the top SNP for resistance to WMV. The CGMMV top SNP belonged to a group of close SNPs in strong LD with the WMV top SNP (Figure 4.A.). It represented broad-spectrum resistance to CGMMV and WMV, which was located only 160 kb upstream of the CMV and CVYV broad spectrum resistance previously described by a common top SNP (Figure 4.A.). The LD between these two broad spectrum resistances was 0.6, so we hypothesized that they were distinct. The hotspot contained 127 genes and the gene distribution among the four categories was almost the same for the four resistances: with around 50 genes having an SNP in LD with the top SNP in a coding sequence (Supplementary Table 5). The CGMMV top SNP was located in the coding sequence of a WRKY transcription factor 55. However, genes within the hotspot were distributed in exactly the same categories for the two broad spectrum resistances except for one (Figure 4.B.), thus questioning our hypothesis of two independent broad-spectrum resistances.

The hotspot on chr 6 for resistance to PRSV and ZYMV (Supplementary Figure 8.I.A.) had a high amount of top SNPs due to the respective presence of three and five QTLs for resistance to PRSV and ZYMV located in this region. We analyzed the LD from the common ZYMV and PRSV top SNP, CucSaCL_Chr6_10946966 (triangle). The two other top SNPs for PRSV resistance, as represented by the two triangles, had respective LDs of 0.6 and 0.02 with CucSaCL_Chr6_10946966, suggesting that there were distinct LD blocks and validating the presence of a cluster. We retrieved 414 annotated genes at this hotspot, but only 140 of them were considered as candidates with non-independent SNPs in their sequences. Only 92 out of 154 candidate genes were distributed in the same categories from QTL_PRSVCH6_01_ and QTL_PRSVCH6_02_, thus contrasting with the hotspot on chr5 (Supplementary Figure 8.I.B.).

The two isolated QTLs on chromosomes 2 and 6 for CMV resistance respectively contained 78 and 127 genes with at least one SNP in LD with the top SNP (Supplementary Table 5 and Supplementary Figure 8.II. and III.).

## Local kinships revealed haplotypes associated with resistance phenotypes and genetic structure

**Figure 5:**
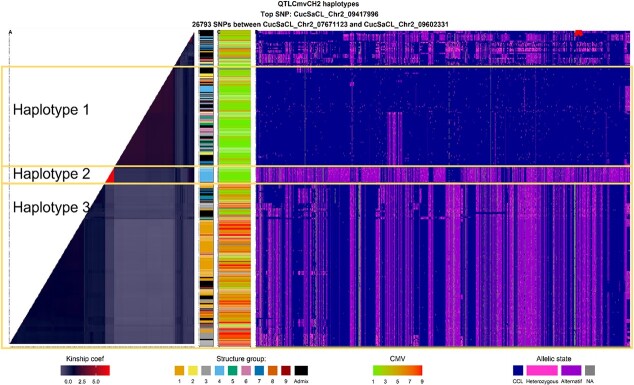
Genetic organization of the QTL_CMVCH2_ (26 793 SNPs between 7.7 Mb and 9.6 Mb) in relation with the genetic structure and level of resistance **A.** Heatmap of the local kinship **B.** Genetic group of accessions (see color code Figure 1). **C.** Resistance level to CMV 1-resistant, 9-susceptible. **D.** Haplotype of each accession. Each SNP is colored according to its allelic state: in blue when the SNP is homozygous as the reference genome CCL, purple for alternative homozygous, pink for heterozygous. The red cross represents the location of the top SNP.

We performed hierarchical clustering of the local kinship at the two resistance hotspots and on the two QTLs for CMV resistance on chr2 and chr6. This revealed several resistant haplotypes unequally distributed among the genetic structure groups. Two resistant haplotypes were found in QTL_CmvCH2_, i.e. one large segment from the reference genome CCL, a CMV resistant source, and one specific to group 4 (Figure 5). We detected several haplotypes in QTL_CMVCh6_ (Supplementary Figure 9.I.). The second haplotype was mainly present in resistant accessions from groups 2 and 4, and admixed accessions. Other haplotypes in QTL_CMVCh2_ and QTL_CMVCh6_ pooled susceptible or resistant accessions due to the presence of other resistant QTLs in the genome.

**Figure 6:**
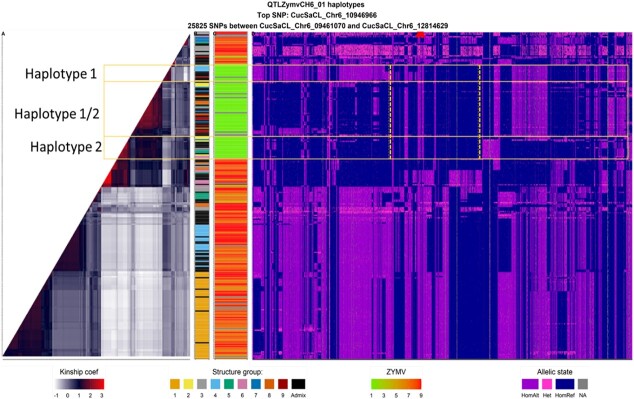
Genetic organization of the hotspot on chr 6 (25 825 SNPs between 9.4 Mb and 12.8 Mb) in relation with the genetic structure and level of resistance **A.** Heatmap of the local kinship **B.** Genetic group of accessions (see color code Figure 1). **C.** Resistance level to ZYMV 1-resistant, 9-susceptible. **D.** Haplotype of each accession. . Each SNP is colored regarding its allelic state: in blue when the SNP is homozygous as the reference genome CCL, purple for alternative homozygous, pink for heterozygous. The red cross represents the location of the top SNP.

For the hotspot on chr5, the local kinship pattern revealed numerous haplotypes (Supplementary Figure 9.B.). Multiple susceptible haplotypes were found at the hotspot on chr6 (Figure 6), including three different ZYMV resistant haplotypes, with one of them possibly resulting from the recombination of the other two (vertical yellow dashed lines). These three ZYMV resistant haplotypes were pooled in one haplotype formed from the five top SNPs (Supplementary Figure 10).

## Discussion

We observed plant responses to inoculation of six virus species in 268 cucumber landraces, elite lines and hybrids. The multi-resistant accessions in this panel were mainly derived from Asian germplasm, i.e. genetic group 2 in this study, as described in the literature [16]. We phenotyped “TMG-1” (from group 2) as resistant to the three potyviruses and to CMV, in line with previous results [21, 31]. We highlighted that “TMG-1” also displayed resistance to CGMMV and CVYV. Interestingly, we observed that the “Cucumber Chinese Long” landrace (also from group 2), displayed better resistance than “TMG-1” to the six studied viruses whereas it was only previously known for its resistance to CMV [9]. The two other accessions widely known to be multi-resistant to potyviruses, “Dina-1” and “02245” [16] were not included in the panel. We also revealed multi-resistance in the slicer groups with the “Sweetslice” landrace (Supplementary Table 3).

## GWAS mapped previously known and new resistance QTLs.

We implemented GWAS analysis to assess the panel response to the six virus species investigated. Mapping of significant QTLs was particularly efficient for monogenic resistance with a medium to large effect (ZYMV, PRSV, CVYV and WMV). It also succeeded in mapping polygenic resistance with medium effect QTLs, despite the marked effect of genetic structure (CMV). For some viruses, such as CGMMV, much of the phenotypic variance remained unexplained, thus suggesting complex determinisms (Figure 2). Mapping six virus resistances by GWAS in a common panel enabled us to highlight a global architecture of resistance to viruses. We mapped 18 loci (Table 2), out of which 15 formed two resistance hotspots in the genome, located on chr 5 and 6. The hotspot on chr 5 remarkably conferred resistance to four different virus families. We identified three broad-spectrum resistance loci nested within these two hotspots, i.e. QTLs sharing a same top SNP or two close top SNPs with high LD, for different virus species. One of these broad-spectrum resistance loci conferred resistance to a cucumovirus (CMV), and an ipomovirus virus (CVYV), while the second one conferred resistance to a tobamovirus (CGMMV) and a potyvirus (WMV), while the last one, on chr6, conferred resistance to two potyviruses (ZYMV and PRSV).

The genetic architecture of cucumber resistance to CMV is complex and still not completely resolved [16]. Only one locus was mapped by segregant analysis from the “02245” accession in linkage group VI and called *cmv6.1* [27]. We found a resistance QTL on chr6 but it was physically located 20 Mb away from *cmv6.1*, thus suggesting the identification of a new CMV resistance locus on chr6. We also identified a QTL on chr2. This QTL contained an EiF4G (Supplementary Table 6), i.e. a gene coding for a protein that belongs to a family well-known for conferringresistance to potyviruses [19] and to CMV in *A. thaliana* [34]. Nevertheless, as no SNP was detected in the EiF4G sequence in this study, we assumed that this gene was not involved in resistance to CMV. More recently, an EiF4E gene was shown to be involved in cucumber CMV resistance by CRISPR-Cas9 [2]. Note that EiF4E was located in one of the minor QTLs for ZYMV on chr1 but did not have any SNP within the EiF4E sequence.

For the potyviruses, the well-known resistance loci mapped for PRSV and ZYMV, i.e. *prsv* and *zym-1* [1, 29], colocalized with the resistance hotspot we mapped on chr6 for PRSV and ZYMV resistance, and also interestingly with *cmv6.1* [27]. Actually, *zym-1* has been shown to exhibit different haplotypes (*zym^TMG-1^*, *zym^Dina-1^*, *zym^A192–18^*) coding for vacuolar sorting protein-associated 4 (VPS4) [25]. We did not detect VPS4 in the last annotation of the reference genome published, but the top SNP detected was located <16 kb from the VPS4 physical position on the previous version of the reference genome (same accession). Moreover, the presence of different VPS4 resistant alleles described in the literature was consistent with our mapping of a cluster of QTLs for resistance to ZYMV: the different top SNPs for ZYMV in the same LD block would indicate different haplotypes conferring the same phenotype rather than different close resistance genes. In accordance, the local kinship findings revealed multiple susceptible haplotypes and three resistant haplotypes (Figure 6). In the literature, ZYMV and PRSV resistances were located 1.2 Mb away (Y. [33]) while we found a common SNP for both viruses. The genes *Prsv* and *zym-1* were described as monogenic in segregating populations, while we found a cluster of QTLs on chr6, which would give the same results as monogenic resistance in segregating population studies. Both resistances were described as recessive and, by contrast, we found an additive allelic interaction. We did not map any WMV resistance on chr6, while a locus was located 19 Mb from PRSV and ZYMV resistance on chr6 [28], probably due to the lack of highly susceptible accessions for this virus in the panel or to the high level of WMV resistance possibly being overestimated in some panel accessions. Overall, these findings confirmed the independence of WMV and PRSV/ZYMV resistance on chr6.

Resistance to CVYV, called *CsCvy-1*, was found in the “CE0749” accession, and mapped by bulk segregant analysis in a 626 kb interval in linkage group V [22]. *CsCvy-1* perfectly colocalized with the hotspot on chr5. QTL_CVYVch5_ was wider than *CsCvy-1* but its top SNP was located within the *CsCvy-1* interval, suggesting that it was the same resistance locus. The overexpression of two RDR1 genes (a and b) could be involved in the broad resistance to CVYV, CMV, CGMMV and ZYMV, whose genes were located 2.5 kb from each other [10, 11]. We observed resistance colocation to CVYV, CMV and CGMMV at the chr5 hotspot. We did not map any ZYMV resistance in this locus but instead we mapped unknown resistance to WMV, another potyvirus. Two RDR1 genes were annotated 8 kb apart at the hotspot on chr5. The second one did not exhibit any SNP while the first one had SNPs but not in significant LD with any of the top SNPs for the resistance to CMV, CVYV, CGMMV and WMV. Patent literature (US8962931B2 and US8779241B2) claims that the number of copies of the RDR1 gene is involved in the resistance to CVYV, CMV, CGMMV and ZYMV. We hypothesized that the number of RDR1s at the hotspot on chr5 was likely involved in resistance to CMV, CVYV, CGMMV and WMV in the panel and our top SNP would be in LD with this copy number. The CGMMV top SNPs mapped in the coding sequence of WRKY transcription factor 55 and this gene family has been shown to be involved in pepper hypersensitive response to the tobacco mosaic virus (TMV) [20]. CGMMV and TMV both belong to the tobamovirus family. The presence of a second resistance gene at this hotspot would be consistent with the local LD analysis findings.

## A panel with an history of selection for resistance

The panel was mainly composed by elite lines from different breeding programs which impacted the genetic structuring and QTL mapping.

First, due to modern selection, the LD extent in the panel (289 kb) was sixfold wider than in non-elite populations of Eurasian cucumbers ( [23]; X. [32]). All QTLs mapped in this study were quite wide (average of 1.6 Mb), suggesting the presence of selective sweeps due to selection and modern introduction of resistance in elite lines of the panel. The hotspot on chr6 was 2 Mb wider than the hotspot on chr5, suggesting a more recent introduction or intense selection. The hotspot on chr 6 has been studied since 1985 and found to control qualitative resistance that is easy to introduce in breeding germplasm [21]. Conversely, the hotspot on chr 5 was first described in 2011 and it controls quantitative resistance [11]. We hence favored the second hypothesis. Therefore, QTL intervals in this elite panel were similar to those of segregating populations, yet QTL mapping by GWAS was still advantageous given that it did not require generation advancement and QTL effects were directly analyzable in diverse genetic backgrounds.

Second, the resistant allele frequencies in the different genetic groups were associated with the added value of resistance for the target market, thereby orienting breeding in favor of resistance introduction. From this perspective, we assessed the origin and contact period with crops of the different viruses according to frequencies of both resistant phenotypes and alleles among genetic groups.

CMV occurs worldwide [13], which could explain the observed multiple resistance sources and resistance QTLs across genetic groups of the panel. By contrast with the resistance to potyviruses, the different CMV resistant sources displayed different resistance loci rather than different haplotypes at the same locus. For instance, genetic group 4 was highly resistant while being homozygous susceptible at the QTL_CMVCH5_ top SNP. QTLs for CMV resistance on chr 2 and 6 were almost the same length, yet the LD decayed faster in QTL_CMVCH6_ than in QTL_CMVCH2_ (Supplementary Figure 8), suggesting a more recent introduction or intense selection of resistance on chr2 than on chr6. The top SNP of QTL_CMVCH2_ was located at the upstream extremity of the LD block, thereby enhancing the possibility of recombination that would break the LD block and enhance genetic diversity to some extent.

Cucumber resistance to potyviruses on chr6 has been successfully introduced in most breeding germplasm. The balanced frequency of resistant alleles and phenotypes across genetic groups, in addition to the major gene effect, explained the high significance levels of ZYMV and PRSV, as highlighted in the Miami plots (Figure 3C). The local LD and kinship studies revealed the presence of different resistant haplotypes, presumably introduced from different sources. A resistant haplotype contained the “TMG” sequence and would be associated with *zym™^G^*, another resistant haplotype pooling the “SweetSlice” landrace and group 4 accessions could be associated with *zym^Dina-1^* or *zym^A192–18^* in the light of the slicer background of “Dina-1” and “A192–18”. The third resistant haplotype might be the result of recombination of the first two resistant haplotypes.

CVYV is close to potyviruses which was initially confined to the Middle East [3]. Its recent spread boosted the resistance demand, especially with regard to the Long-Dutch type pooled here in genetic group 1. Group 1 accessions displayed the same intermediate level of resistance as group 9 accessions that come from the area of origin of CVYV. Group 1 non-specific alleles came more from the group 9 than from group 2 (Figure 1). However, haplotype studies pooled resistant group 1 accessions with group 2 accessions rather than group 9 accessions. Therefore, CVYV resistance in group 1 probably resulted from partial introduction of total resistance from the resistant group 2 landrace rather than the intermediate resistance of group 9 that has yet to be mapped. CVYV resistance was associated with the hotspot of resistance to CMV, WMV and CGMMV on chr5 (Table 2), with these last resistances being potentially the result of CVYV resistance introduction. Accordingly, we hypothesize that the WMV resistance on chr5 was specific to the intermediate resistance of the group 1. However, the intermediate control of WMV displayed resistant phenotypes and then WMV results should be questioned accordingly.

CGMMV has only become a major threat in cucumber glasshouse crops in recent years [14]. Therefore, similar to CVYV resistance, CGMMV resistance was less influenced by modern breeding than resistance to potyviruses and CMV. Total resistance to CGMMV was only found in Asian genetic group 2 landraces in the panel (Figure 1). The low number of accessions in this group and the absence of other highly resistant accessions hampered mapping for resistant alleles that were not introduced in elite germplasm. The resistance seemed to be highly polygenic (Figure 2) as the part of the PV explained was substantial despite a top SNP low effect. Further QTLs thus remain to be mapped using a population larger than the panel studied here.

## Prospects

Here we were able to map monogenic resistance for ZYMV, PRSV, WMV and CVYV and multiple quantitative resistances for CMV and CGMMV via GWAS. The development of breeding markers to capture these multiple genes/haplotypes should take the genetic background into account. Genome-wide selection (GWS) is a statistical method that relies on identical datasets but aims to predict a phenotype instead of mapping QTLs. GWS successfully improved the resistance level of a structured *Cacao* population to *Moniliophtora* spp., whereas MAS from GWAS QTLs could not [17]. GWS could hence be an interesting solution against viruses with several low effect QTLs. For CVYV and CGMMV, only one and two significant SNPs were mapped by GWAS while a major part of phenotypic variance was still explained by genetic (Figure 2). Resistance to these two viruses, in addition to CMV and its complex genetic architecture, are candidates for implementing genome-wide selection for virus resistance in cucumber.

## Materials and method

 

## Panel assembly, genetic structure and kinship

The panel pooled around 200 elite lines and 50 landraces to broaden the genetic diversity. Several hybrids resulting from the cross of elite lines from the panel were included in phenotypic studies to enhance statistical control of the environmental effects on phenotypes and study allelic interactions. Genotypes were derived from whole genome resequencing via Illumina 150 bp pair-end sequencing to 20x coverage. GATK Best Practices were used for SNP calling and the final variant callset was hard-filtered for SNPs with a missing rate under 10%, a heterozygous rate under 15% and a minor allele frequency (MAF) above 3125%) are given in the Supplementary Material 1. It resulted in an SNP matrix called M_NA10_ containing 258 lines and 1 174 509 SNPs. We estimated the LD extent by randomly selecting 10 K SNPs on each chromosome. We calculated the r^2^ between all SNPs in a 1 Mb window and performed loess regression with a span of 0.2. The LD extent was assessed when r^2^ = 0.2 was reached.

The population genetic structure matrix was designed via the sparse nonnegative matrix factorization (sNMF) procedure developed by [7]. The algorithm was run on M_NA10_ using one to twenty putative genetic groups and 50 iterations per number of groups.

The kinship matrix was estimated with the following equation [4]:

[1] \begin{align*}A=\frac{WW^{\prime }}{c},\ \textrm{where} \end{align*}

${W}_{ik}={X}_{ik}+1-2{p}_k$ , with i being the individual and k the SNP.

X is the genotype matrix.

p_k_ is the frequency of the allele 1 at SNP k

$$ \begin{align*} c=2{\sum}_k{p}_k\left(1-{p}_k\right) \end{align*}$$

## Inoculation, symptom severity scoring and phenotype analyses

The panel phenotypic response to the six viruses was evaluated with three randomized complete blocks of five plants per accession after mechanical inoculation of CMV, CVYV, WMV, PRSV and ZYMV and four plants per accession for CGMMV. Each virus was tested in an independent trial in insect-proof glasshouses, then no confirmation tests for virus identity were performed. Details concerning inoculation are given in Supplementary Material 2.

The symptom severity (SS) was individually scored around 14 days post-inoculation (DPI) when 100% of intermediate and susceptible checks revealed the expected symptoms. We used the following notation scale: 1 – no symptoms, 3 – one to three symptom spots on one leaf, 5 – limited symptoms areas on young leaves, 7 – more numerous symptoms on young and old leaves, 9 – plant growth halted, development of obvious severe symptoms (leaf mosaic, vein yellowing, deformation, bubbling depending on the virus). Even scores were attributed when plants displayed intermediate symptoms between uneven scores. All scores higher or lower than 2 standard deviations (SD) within each accession were considered outliers and discarded from the phenotypic dataset.

We estimated the experimental heritability from Equation 2 to assess the phenotyping quality and trial repeatability. Experimental effects were corrected by including two fixed effects in the model: the location within the trial (block) and the quantity of inoculum received by the plant (repetition).

[2] \begin{equation*} y= W\nu + Gg+e, \end{equation*}

where **y** is the vector of observed symptom severity values per plant,

ν is a fixed effect matrix (block and repetition),

**W** is the fixed effect incidence matrix

**g** is the vector of random accession effects with distribution

$$ N\left(0,I{\sigma}_g^2\right). $$

**G** is the accession incidence matrix.

**e** is the vector of errors with distribution $N\Big(0,I{\sigma}_e^2\Big).$

The experimental heritability from ${\sigma}_g^2$ and ${\sigma}_e^2$, with N_block_ = 3 and N_rep_ = 4 or 5

[3] \begin{align*} {h}^2=\frac{\sigma_g^2}{\sigma_g^2+\frac{\sigma_e^2}{N_{block}\times {N}_{rep}}} \end{align*}

As symptom severity scores were discontinuous, a generalized Poisson mixed linear model [Equation 4] was used to extract continuous BLUPs. These BLUPs were called PoPs. The glmer function from the R package lme4 was used to perform this transformation.

[4] \begin{align*} {y}_{brg}\to P\left({\lambda}_{brg}\right) \end{align*}

Where ${y}_{brg}$ are the raw phenotypes following a Poisson law (P) of parameter ${\lambda}_{gbr}$

with log(${\lambda}_{brg}$) = $W{\nu}_{br}+ Gg$

**where W** is an incidence matrix associating fixed effects (block and repetition) per plant

**ν** is a fixed effect vector,

**G** is an incidence matrix associating the accession effects with plant performance **g** is a vector of random accession effects with an assumed distribution of

\begin{align*} N\left(0,I{\sigma}_g^2\right). \end{align*}

## GWAS

Due to seed stock and/or germination issues, the number of accessions tested per trial ranged from 240 to 250 (Table 1). In that setting, one genotype matrix per virus was derived from M_NA10_ by removing SNPs with at least one NA and with MAF < 3.1% (i.e. when there were less than 12 homozygous accessions in the least frequent genotypic class), resulting in six M_NA00_, called M_NA00_CMV_, M_NA00_CVYV_, M_NA00_CGMMV_, M_NA00_WMV_, M_NA00ZYMV_ and M_NA00_PRSV_, these matrices contained at least 1 SNP every 500 bp (Supplementary Table 4).

The MLMM model described in [26] and implemented in the R package mlmm was used to perform GWAS. MLMM is a multistep approach, with each step being a GWAS using the MLM described below but complemented with each top SNP of previous steps included in the model as fixed effects. Five models were tested, i.e. kinship (K), genetic structure (Q5), genetic structure (Q9), kinship + genetic structure (KQ5), and kinship + genetic structure (KQ9). We applied MLMM on PoPs using the model:

[5] \begin{equation*} \mathrm{y}={\mathrm{X}}_{\mathrm{M}}\upbeta +{\mathrm{X}}_{\mathrm{W}}\upnu +\mathrm{g}+\mathrm{e} \end{equation*}

where y is the phenotype vector (PoPs)

β is the vector of allelic effects

${\mathrm{X}}_{\mathrm{M}}$ is the genotypic matrix of all lines at the SNP being tested

ν is the fixed effect vector, including the population genetic structure and top SNP depending on the model and MLMM step.

${\mathrm{X}}_{\mathrm{W}}$ is the fixed effect incidence matrix

g is the random genotype effect vector and $\mathrm{g}\to \mathrm{N}\Big(0,{\mathrm{K}\upsigma}_{\mathrm{g}}^2\Big)$

K is the kinship matrix (= I for Q5 and Q9 models) and ${\upsigma}_{\mathrm{g}}^2$ is the genetic variance

e is the error vector, ${\sigma}_e^2$ is the residual variance and $\mathrm{e}\to \mathrm{N}\Big(0,{\mathrm{I}\upsigma}_{\mathrm{e}}^2\Big)$

g and e are assumed to be independent.

The last MLMM step was defined when no further SNPs were found below the Bonferroni threshold or when no phenotypic variance was under genetic control. A Bonferroni significance threshold was set up with a genome-wide error risk of α = 0.05 corrected by the number of independent SNPs previously determined with Gao’s method using a 100 kb sliding window [8]. It estimated 115 391 independent SNPs. Consequently, the Bonferroni threshold was set for α = 0.05 at 4.3x10^−7^ (−log_10_ = 6,4) and for α = 0.01 at 8.7x10^−7^ (−log_10_ = 7,1) for GWAS analyses.

The different implemented models were compared via QQ plots generated from the qqman R package [30]. The null model with only structure effects was also tested to estimate the part of the phenotypic variance explained by genetic groups.

## Post-GWAS analyses

Post-GWAS analyses were performed with the M_NA10_ genotype matrix, *p*-values extracted from the first step of the KQ9 MLMM models and PoPs.

We estimated the part of phenotypic variance explained by the different top SNPs. The effect of the other fixed effects, i.e. the top SNPs, were estimated by subtracting the part of the variance explained by the genetic structure, calculated on the null model, from the part of the variance explained by all fixed effects in the model in which they were studied. These results were studied in parallel to the resistant allele distribution among genetic structure groups. Allelic interactions were assessed only in crosses resulting in heterozygous progenies at the top SNP.

The LD corrected by the structure was calculated for the top SNPs and all physically close SNPs using a 100 kb sliding window [15]. QTL intervals were set when no SNPs in LD with the top SNP (r^2^S_TopSNP)_ > 0.2) were found in the 100 kb sliding window. All genes located in QTLs were extracted from the gff file from the reference genome “CCL” V3 available on the CUGenDB database [6]. We checked for the presence of SNPs in coding and non-coding sequences of each gene and for each SNP we calculated the r^2^ with the top SNP. Kinship coefficients were calculated for each QTL on the M_NA10_ narrowed to the SNP included in the QTL. The local kinship matrix obtained was clustered by hierarchical clustering. Haplotypes were defined based on clustering of the local kinship coefficients to pool resistant lines by potential resistance source.

## Acknowledgments

We would like to thank all the staff who make possible these massive trials: John Woutersen, Ton Kruiswijk, Arien de Koning, Bernadette Cucarella and Stéphanie Gimenez for virus phenotyping and Tommy Stekelenburg, Marieke van Groesen-Hop, Bram Rozier, Zelida Duarte and David Farkas for trial preparation and data management. We also thank David Menley for its English review of the manuscript. We thank the R community for playing the game of open-source scripts and particularly Vincent Segura (MLMM), Eric Frichot and Olivier François (LEA), David Desrousseaux et Brigitte Mangin (LDcorSV), Jeffrey Endelman (rrBLUP) and Xiaoyi Gao (simpleM) and more generally to all contributors to R Q&A platforms.

## Author Contributions

Experiment design: MC and SM, phenotyping: SM, MVH and AR, sequence analysis/genotyping: RL, code and analyses: SM and TMH, writing—original draft preparation, SM, TMH, LM and NB; writing—review, MC. All authors have read and agreed to the published version of the manuscript.

## Data avaibility

The SNP matrix is available on https://zenodo.org/record/6962598, DOI: 10.5281/zenodo.6962598

## Conflicts of Interest

This research was funded by Bayer in the context of a CIFRE PhD

## Supplementary data

Supplementary data is available at *Horticulture Research * online.

## Supplementary Material

Web_Material_uhac184
