# Supplementary material for: Unravelling cucumber resistance to several viruses via genome-wide association studies highlighted resistance hotspots and new QTLs
Source: Hortic Res. 2022 Aug 25;9:uhac184. doi: 10.1093/hr/uhac184 (PMC9627523; doi:10.1093/hr/uhac184)
Supplement: Web_Material_uhac184 [file web_material_uhac184.zip › Article_GWAS_CUC_virus_SupFigs_Rev_round1.docx]

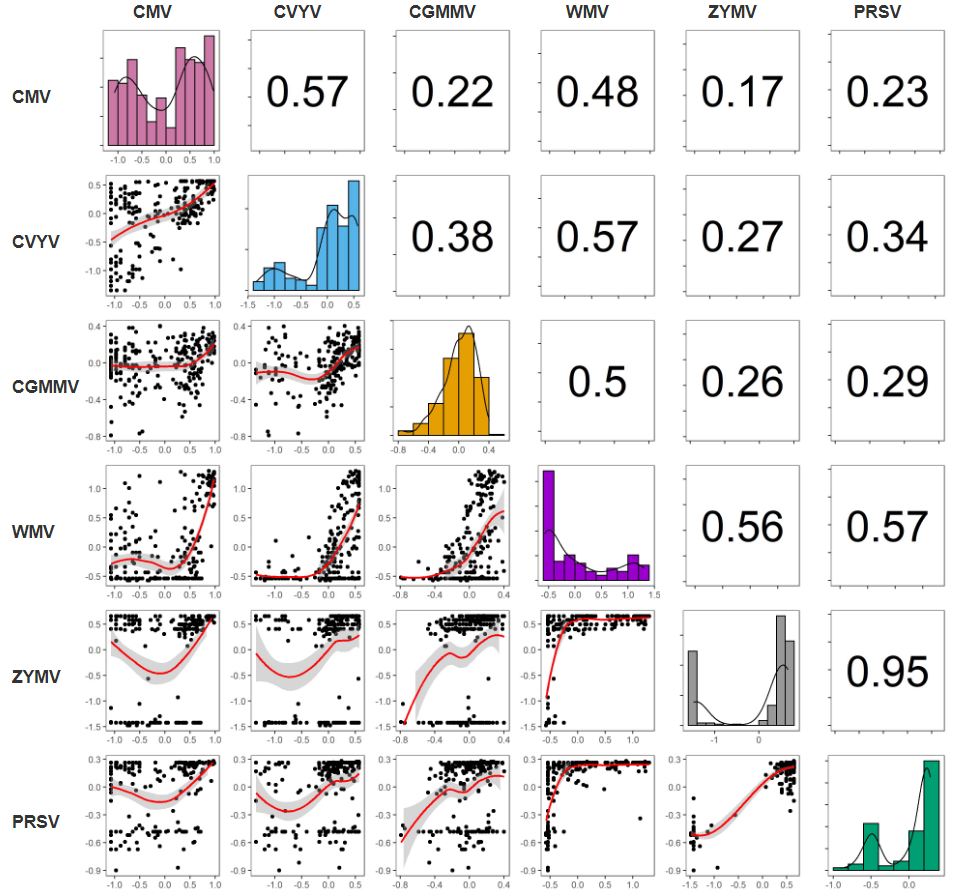


**Supplementary figure 1:** On the diagonal, PoP distribution for CMV, CVYV, CGMMV, WMV, ZYMV and PRSV. Upper part, Pearson’s correlation r^2^, lower, matching scatter plots with the regression curve in red.


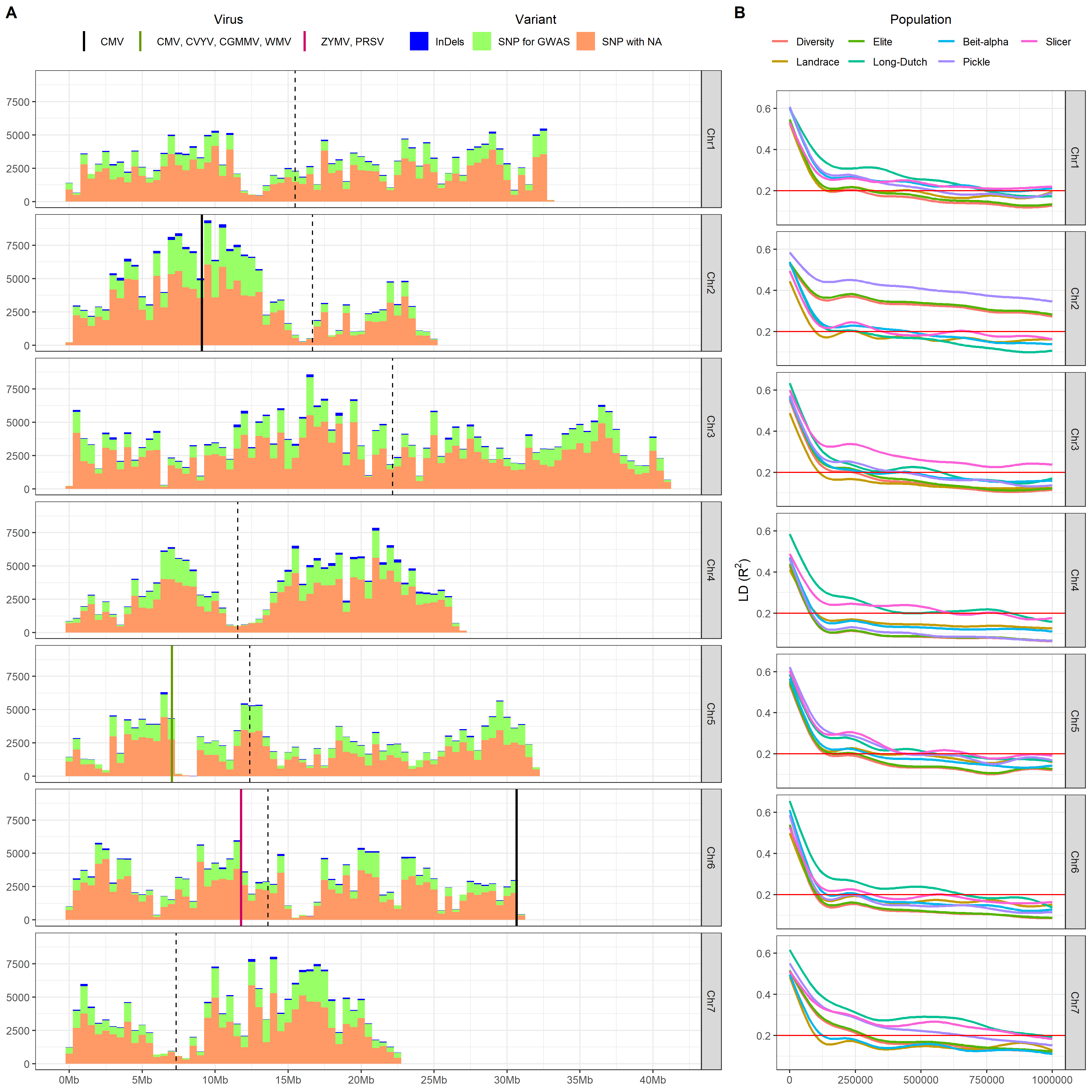


**Supplementary figure 2:** A. SNPs and InDels densities on each cucumber chromosome. Each chromosome contained at least one low SNP density area more or less distant from the centromeric position provided by Li et al. (2019) and represented by vertical dashed lines. There were a larger number of SNPs with at least one missing data (in orange) than SNPs without missing data which were used to run GWAS (in green). Indels were scarce and no enrichment was detected in the direct proximity of the QTLs mapped, represented by the full vertical lines. B. LD decay for each chromosome and subpopulation. The LD extent was assessed when the loess regression first reach an independency threshold of 0.2 (horizontal red line).

**
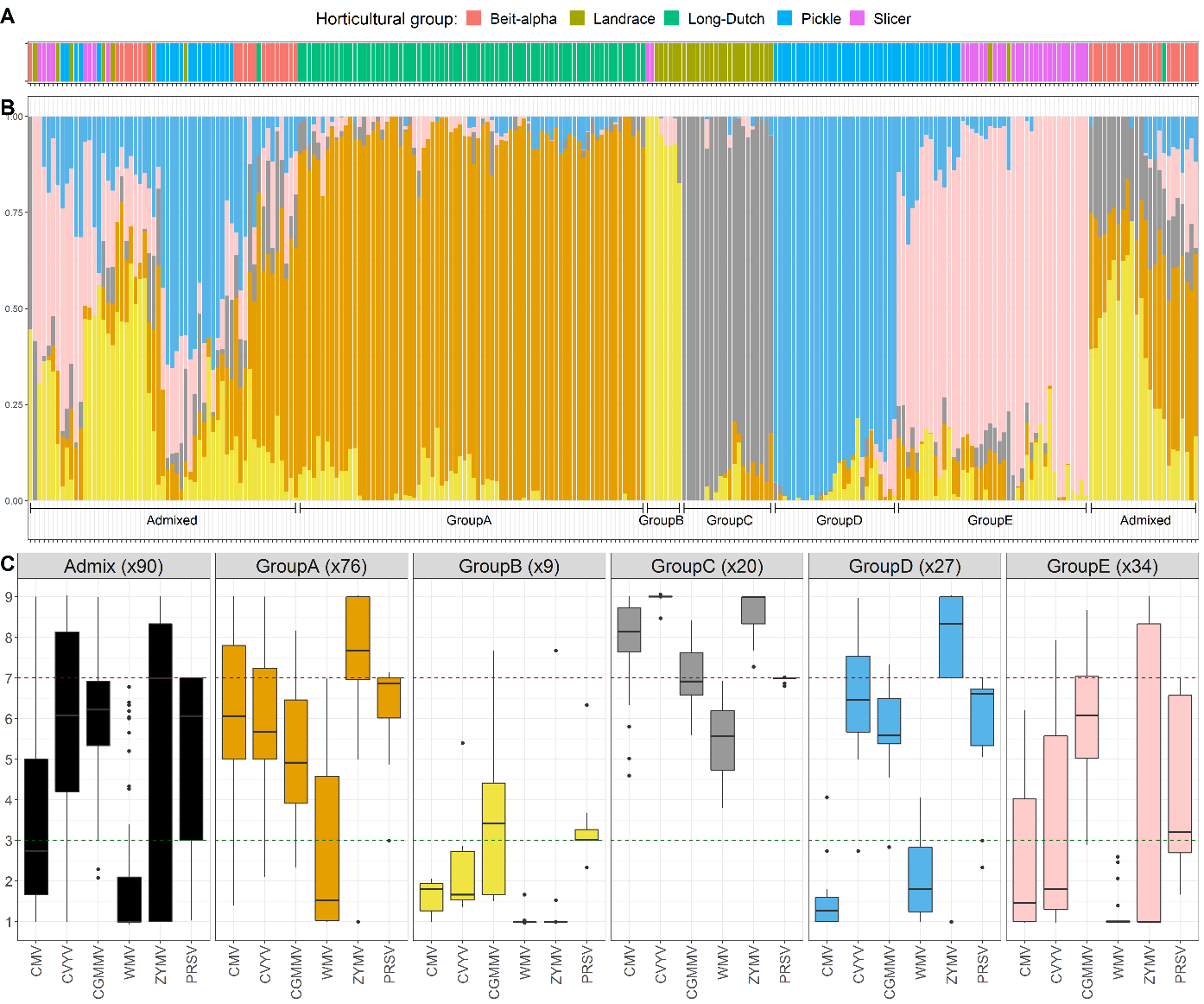
**

**Supplementary figure 3:** Organization of the 256 cucumber accessions across five genetic groups **A.** Each color represented the horticultural group (when known) of the accession. **B.** Barplot representing the percentage attribution of 256 cucumber accessions to nine genetic groups. Accessions were rarely attributed at 100% to one genetic group and the admixture observed results from crosses between breeding programs. Values were extracted with the snmf R package. Genetic groups were consistent with horticultural groups. **C.** Boxplot representing the phenotypic variance in the diversity panel for each virus. Accessions were assigned to a genetic group when the contribution of this group was above 0.6, if no group reached this threshold, the accession was considered as admixed (in black). The number of accessions per group is indicated in brackets.


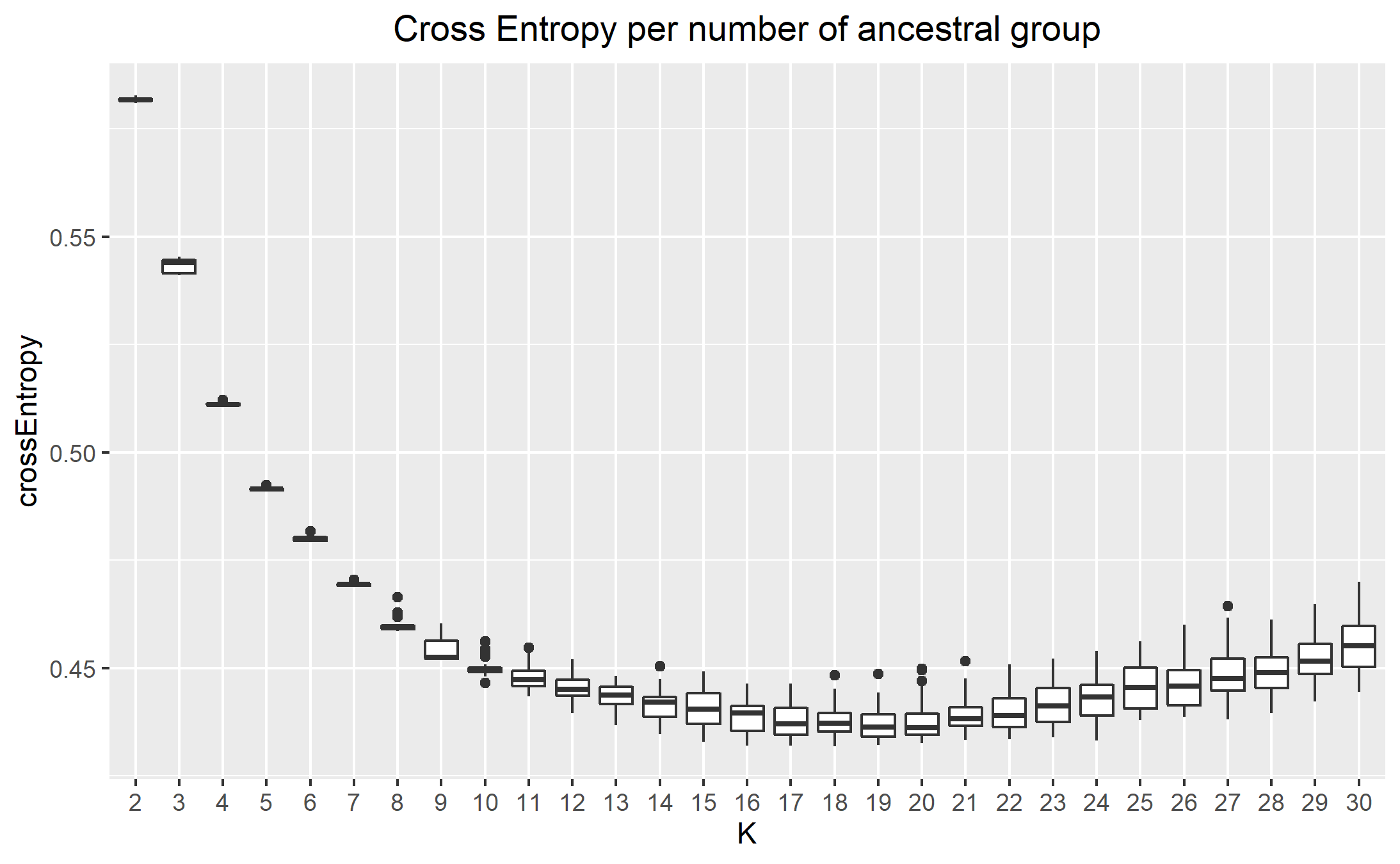


**Supplementary figure 4:** Cross-entropy variation among the 50 snmf iterations for K=2 to K=30. In genetic structure analyses via snmf, the cross-entropy evaluates the fit of a model with K groups by measuring the difference between two of its predictions with different subset of masked genotypes. A smaller cross-entropy reflects a better run regarding the prediction capacity. In this study, the cross-entropy selection criterion regularly decreased from K=2 to K=17, the latest having the lowest entropy criterion.


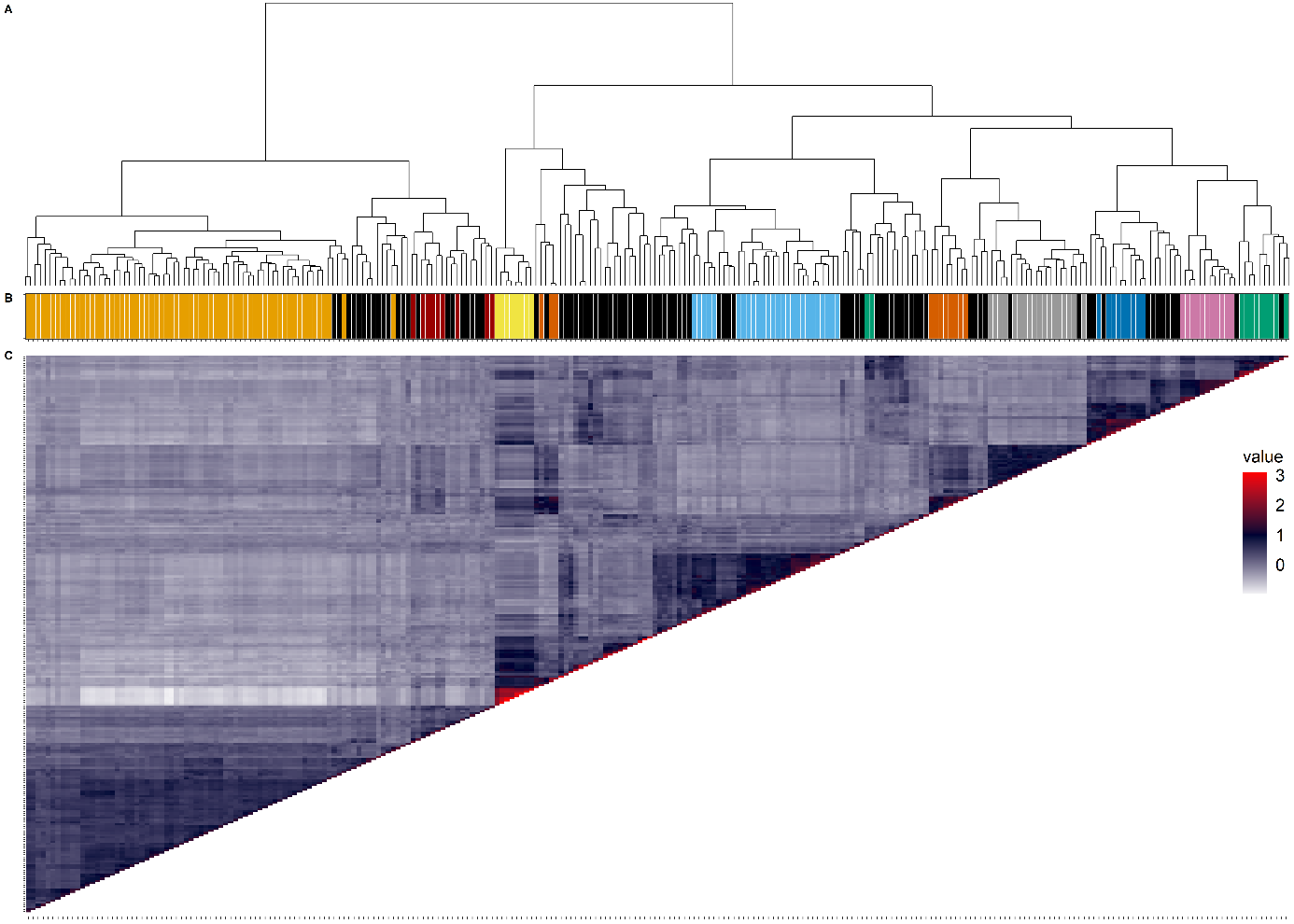


**Supplementary figure 5:** Genetic proximities between the 256 cucumber accessions **A.** Dendrogram obtained from the clustering of the Vanraden additive kinship matrix. **B.** Genetic structure group of the 256 accessions from the snmf analysis (same color code than **Figure 1**). Kinship was consistent with structure group in most cases. **C.** Heatmap of the upper triangle kinship matrix. Darker color spots highlighted clustering of genetically similar individuals. Landraces from group 2 (yellow) and group 3 (grey) displayed higher kinship coefficients than other groups.


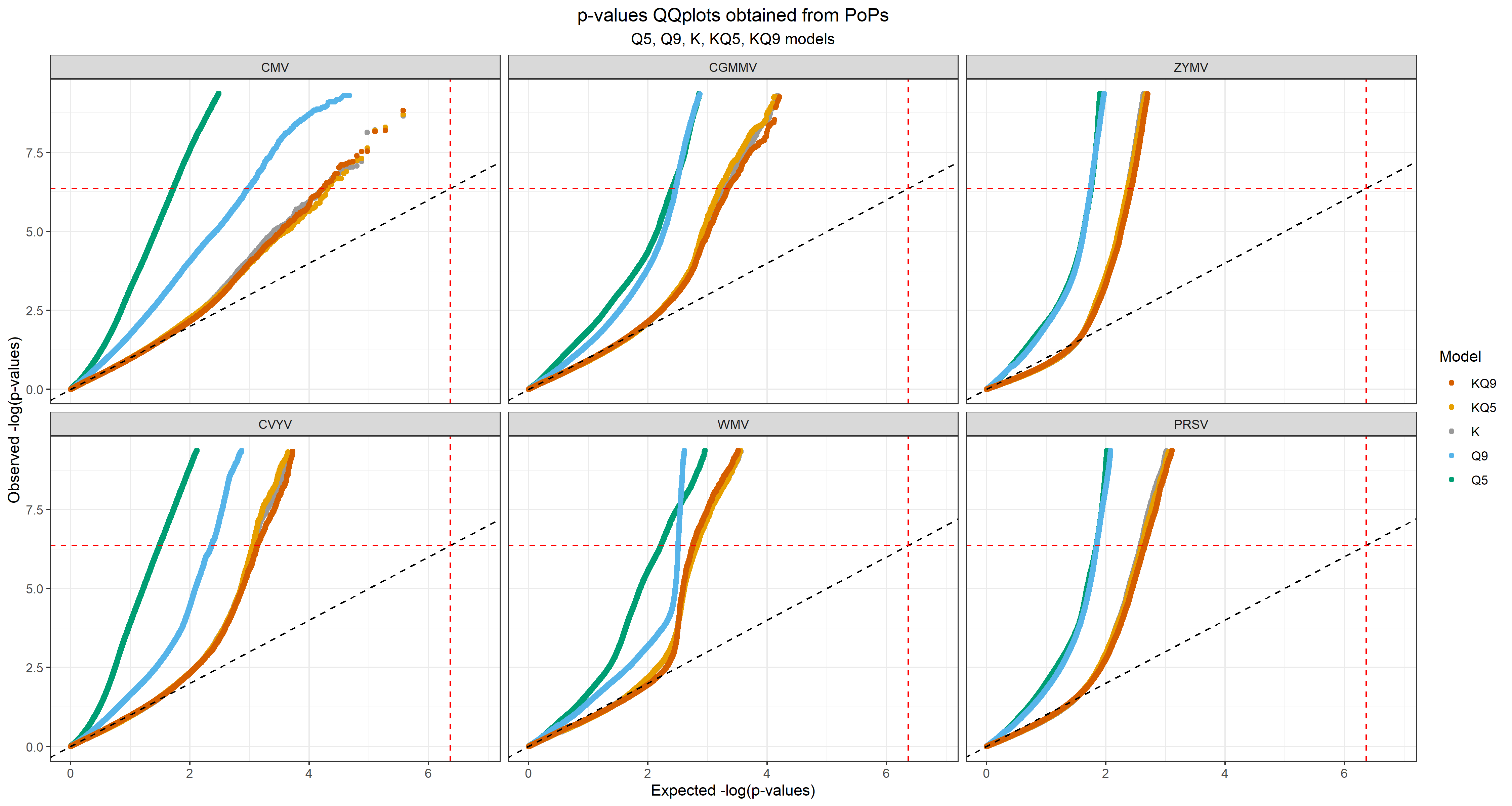


**Supplementary figure 6:** Qqplots for five models tested on the two phenotypes for each virus. P-values are expected to follow a uniform distribution, represented by the bisector between the observed p-values collected by GWAS and the expected p-values simulated under a uniform distribution. The KQ9 model respected the best this hypothesis for all viruses. On the contrary, models without kinship correction have a high excess of low *p*-values.


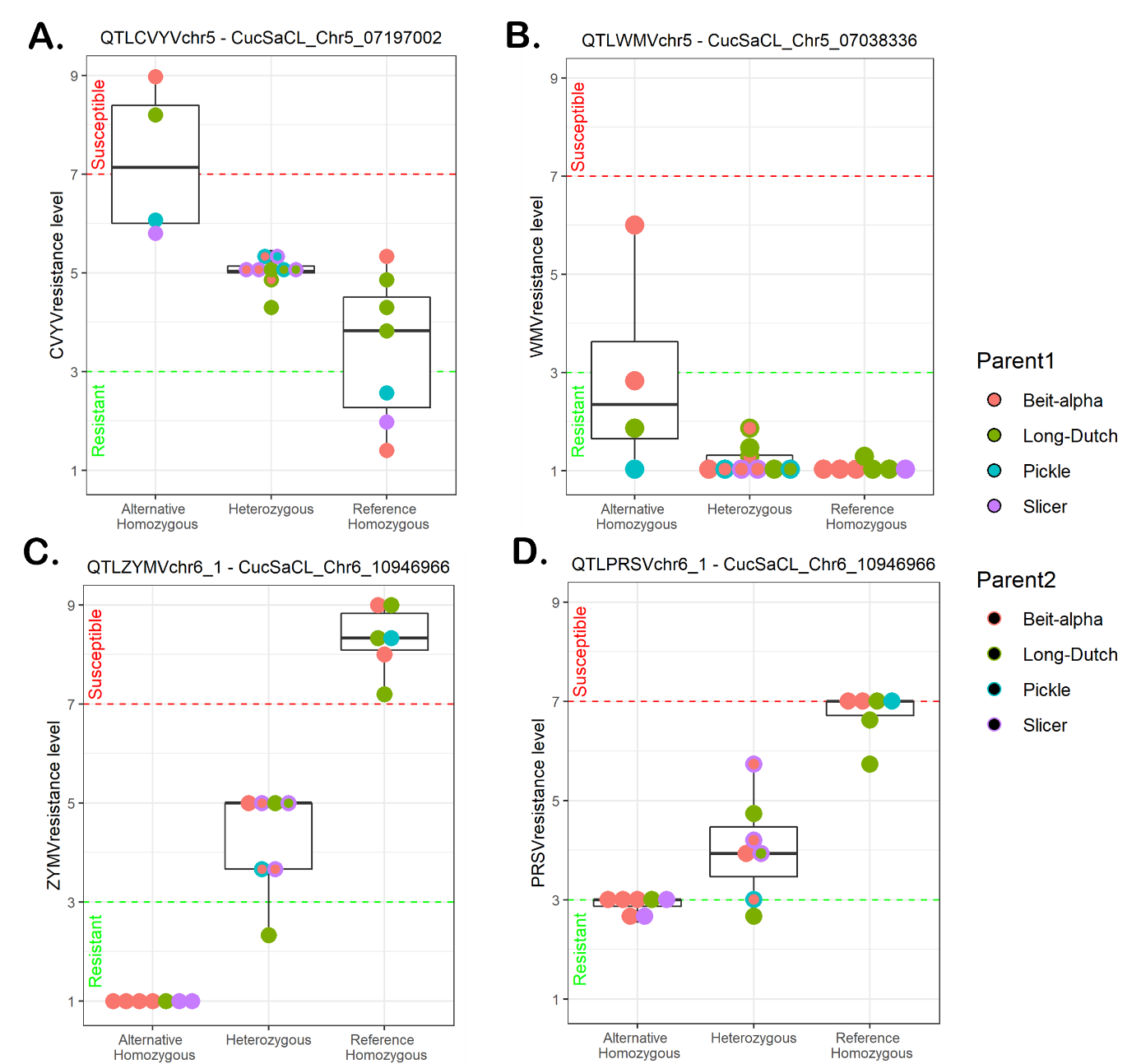


**Supplementary figure 7:** Resistance level of parental lines and derived F1. Heterozygous F1 in hot spots on chr 5 and/or chr 6 were selected as well as their parents and their severity scores were plotted in the Y axis. **A.** Hot spot on chr5 and CVYV resistance. **B.** Hot spot on chr5 and WMV resistance. **C.** Hot spot on chr5 and ZYMV resistance. **D.** Hot spot on chr5 and PRSV resistance.

I.


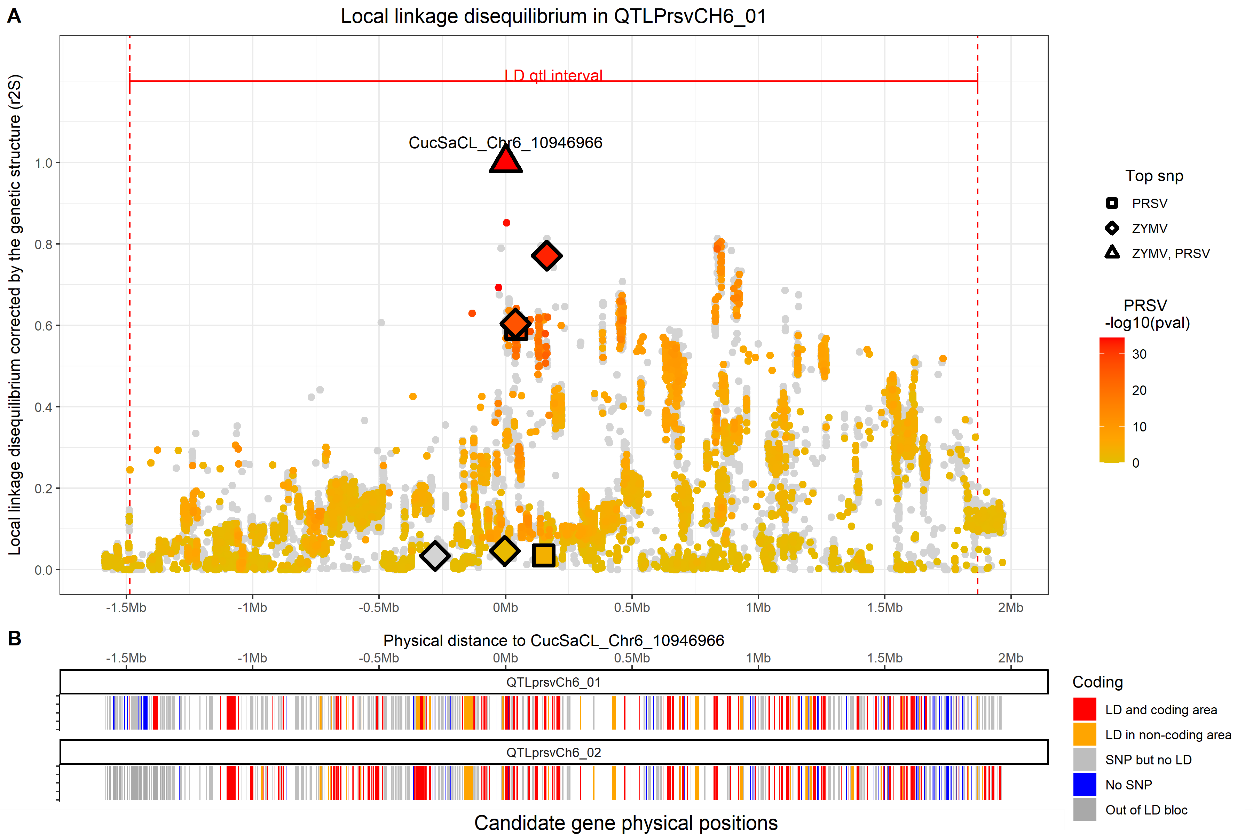


II.


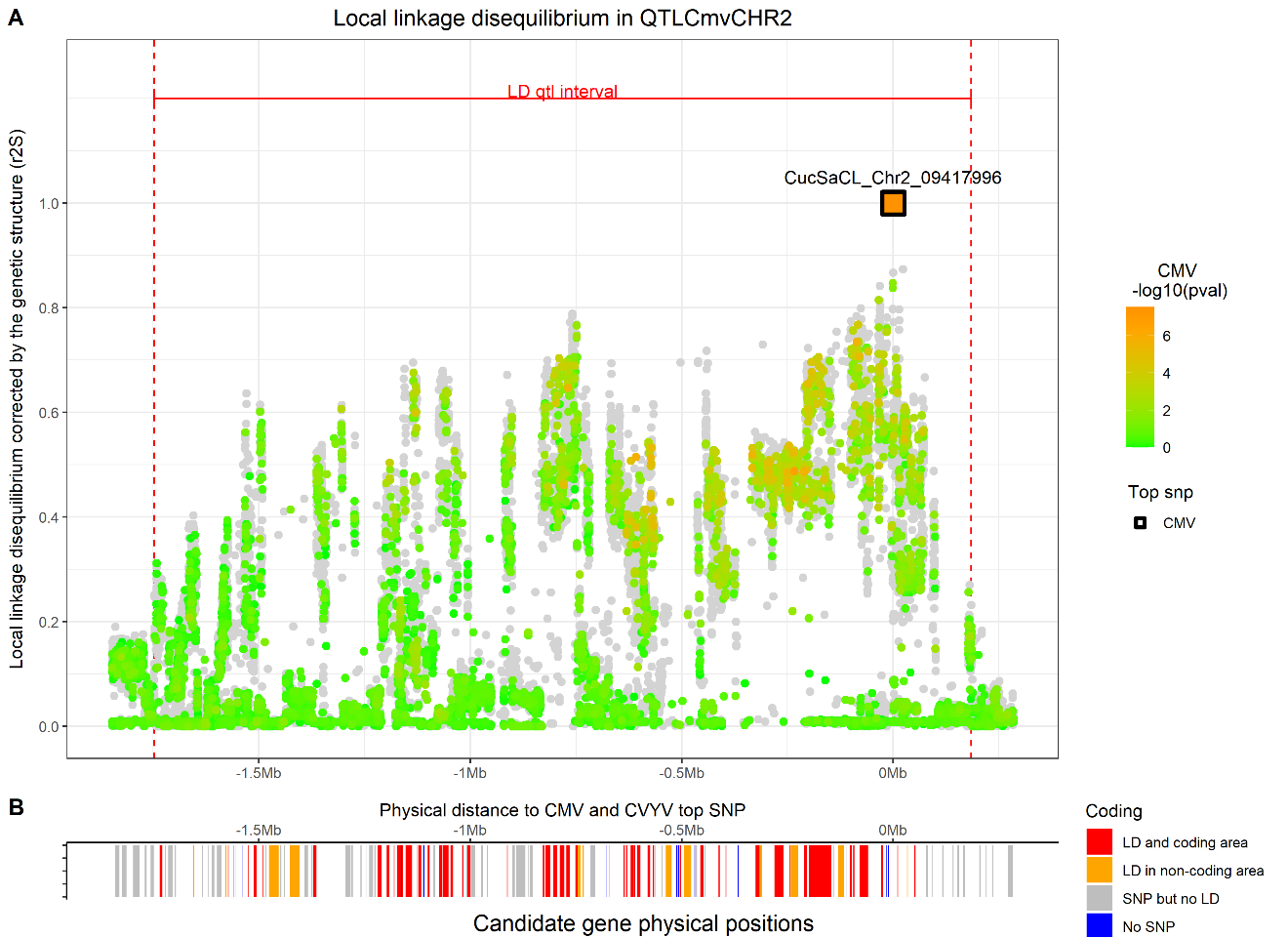


III.


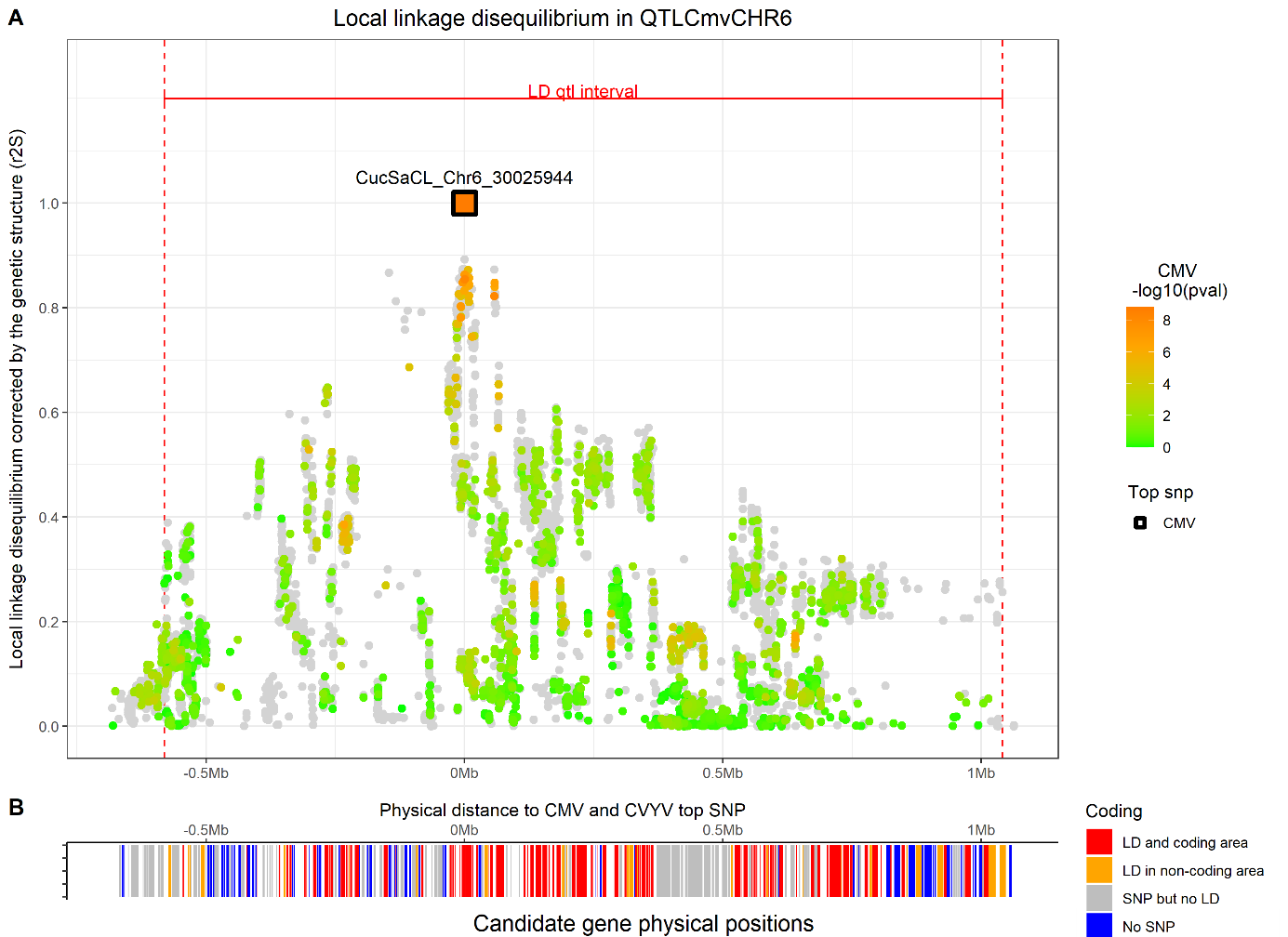


**Supplementary figure 8:** Representation of the local LD and candidate genes in I/ hot spot on chr 6, II/ isolated QTL for resistance to CMV on chr6, III/ isolated QTL for CMV resistance on chr 2. **A.** Representation of the LD between the top SNP and all other SNPs within the QTL. X-axis represents the physical distance from the top, Y-axis the r^2^ corrected by the structure between SNPs and the top SNP. Each dot represents a SNP which is colored according the p-value in the first step of MLMM GWAS for a given virus. Dots were grey when the SNP was not tested by GWAS due to missing data. Dot shape represents the top SNP of the QTL or top SNPs from other resistance QTLs. The QTL interval is delimited by the red dashed line. **B.** Genes located in the QTL, candidate genes colored in red (at least one SNP in LD with the top SNP in a coding area), potential candidate genes colored in orange (at least one SNP in LD with the top SNP in a non-coding area), and other genes in grey or blue.


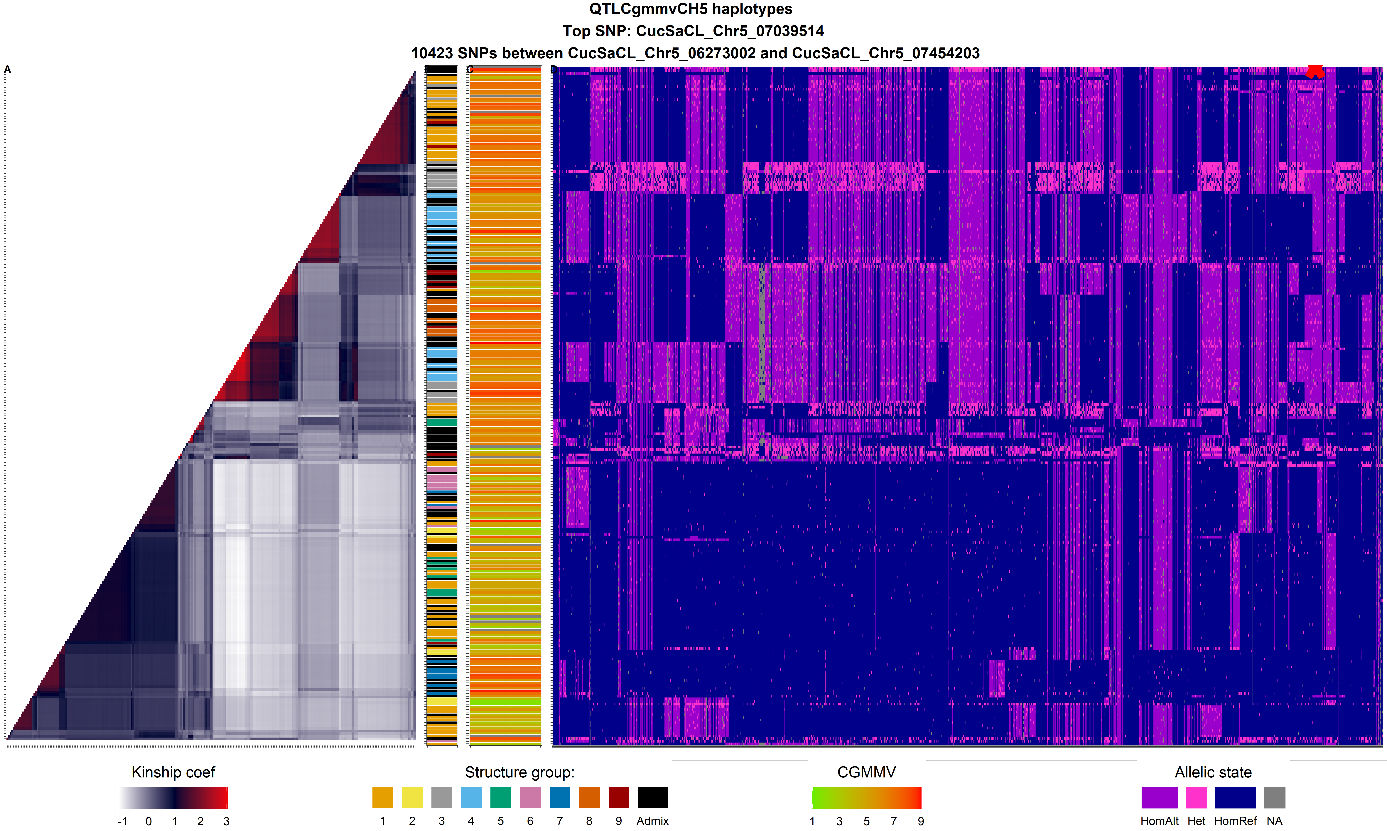

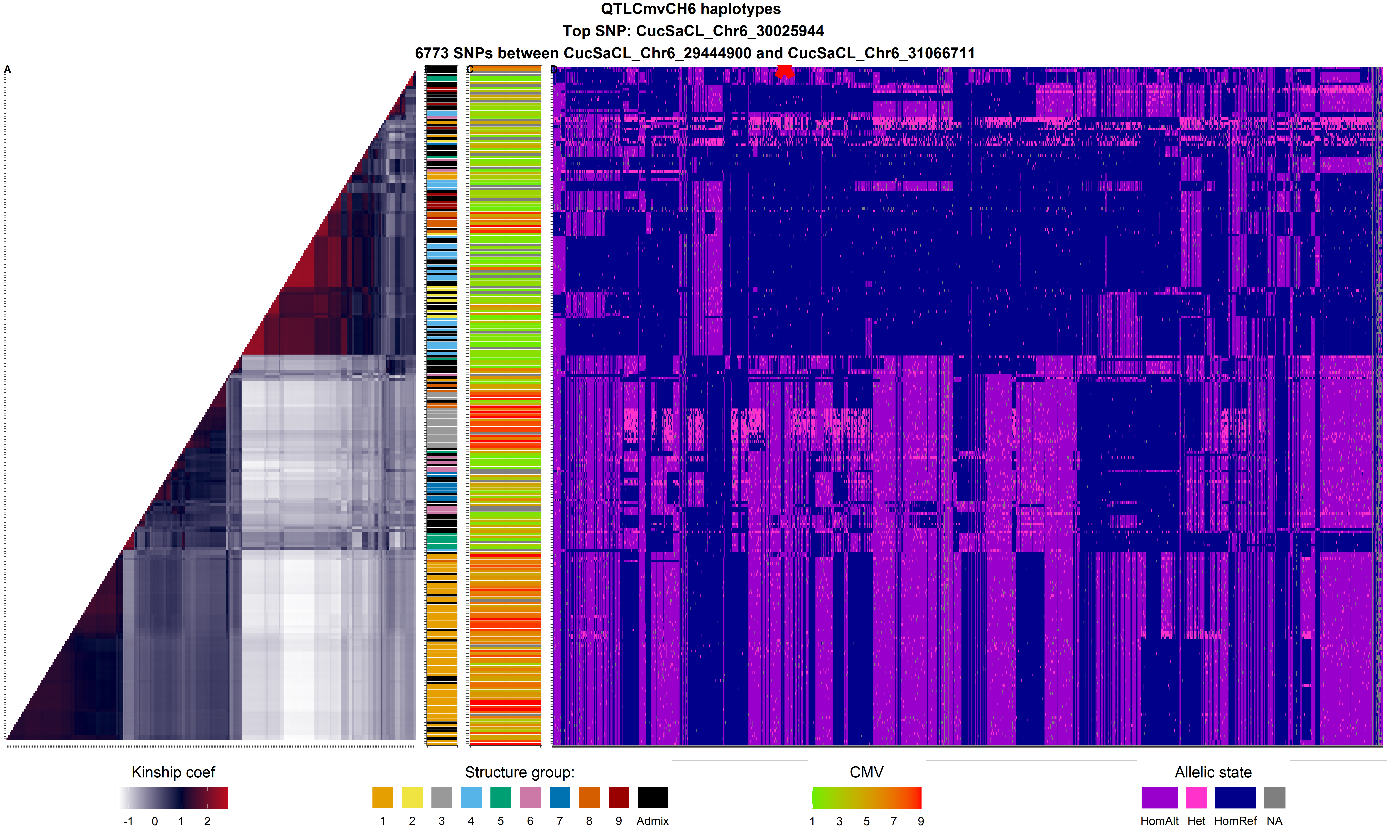


**Supplementary figure 9:** Genetic organization of I/ the hot spot on chr 5 and II/ QTL_CMVCH6_ in relation with genetic structure and level of resistance **A.** Heatmap of the local kinship **B.** Genetic group of accessions (cf color code Figure 1). **C.** Resistance level to I/ CVYV and II/ CMV 1-resistant, 9-susceptible. **D.** Haplotype of each accession. Blue when the SNP is homozygous as the reference genome CCL, purple for alternative homozygous, pink for heterozygous. The red cross represents the location of the top SNP.


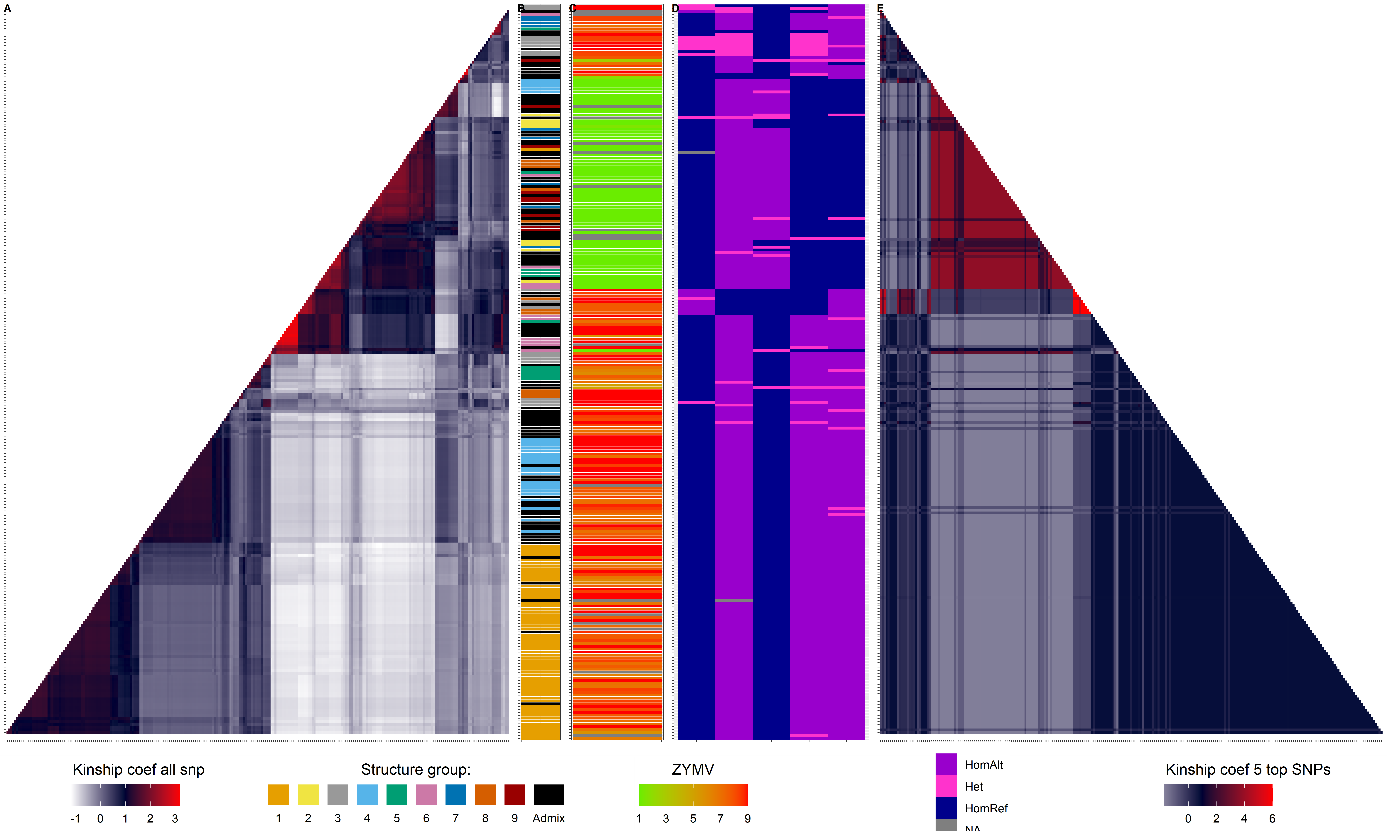


Supplementary figure 10: Genetic organization of the hotspot on chr6 summarized by the five top SNPs selected by MLMM for ZYMV resistance

**Supplementary material 1:** Whole genome resequencing and variant calling

Leaf samples from three plants were bulked for each elite line and landrace and sequenced with Illumina 150 bp pair-end to 20x coverage. The Cornell Chinese Long v3.0 public genome (Li et al. 2019) was used for sequence alignment and variant calling. Variant discovery performed using GATK4 Best Practices (Van der Auwera et O’Connor 2020), first by calling individual sample SNPs, followed by joint genotyping on the population. All biallelic and multiallelic SNPs, as well as InDels, with QD (Quality by Depth) > 18 were collected, resulting in a genetic dataset of 2,081,122 biallelic SNPs, 28,162 multiallelic SNPs and 30,557 InDels. SNPs exhibiting a missing rate above 10%, a heterozygous rate above 15% and a minor allele frequency (MAF) under 3,125% (~8 homozygous genotypes), were discarded. InDels and multi-allelic SNPs were also discarded. Four lines, including three landraces, exhibiting more than 30% missing data and 30% heterozygous rate were discarded from the diversity panel.

**Supplementary material 2:** Virus inoculation protocol

CMV, CVYV, WMV and PRSV trials fit in a single greenhouse compartment while the ZYMV trial was conducted in two growth chambers. CGMMV is a quarantine pathogen in Europe which requires a specific testing protocol, hence it was tested in three different greenhouse compartments, one bloc per compartment. CMV, CVYV and CGMMV trials were conducted in 2019 in Bergschenhoek, the Netherlands, while WMV, PRSV and ZYMV trials were conducted in 2019 in Saint-Andiol, France (Table 1).

WMV, PRSV and ZYMV isolates were separately and mechanically inoculated in different plants of the ‘Diamant F1’ squash variety ten days before the diversity panel inoculation. The CMV, CVYV and CGMMV isolates were separately and mechanically inoculated in different plants of the ‘Corona’ cucumber variety ten days before the diversity panel inoculation. For CMV, two isolates (1:1 concentration) were co-inoculated with one strain developing symptoms in cold conditions and the other one in warm conditions to ensure symptom observation. When cotyledons were fully expanded, around one-week post-sowing, 10g of Diamant or Corona leaves were mixed in 50 ml of phosphate buffer Na_2_HPO_4_-0.03M + Sodium diethyldithiocarbamate 0.2%, and 2.5g of charcoal and carborundum. Inoculums were kept in ice during inoculation (4°C) and cotyledons were hand-rubbed twice, by two different operators to avoid inoculation escape. Inoculum was refreshed between each genotype, resulting in a potential inoculation of more viruses in the first plant of each repetition.
