# Supplementary figures and images for: Unravelling cucumber resistance to several viruses via genome-wide association studies highlighted resistance hotspots and new QTLs
Source: Hortic Res. 2022 Aug 25;9:uhac184. doi: 10.1093/hr/uhac184 (PMC9627523; doi:10.1093/hr/uhac184)

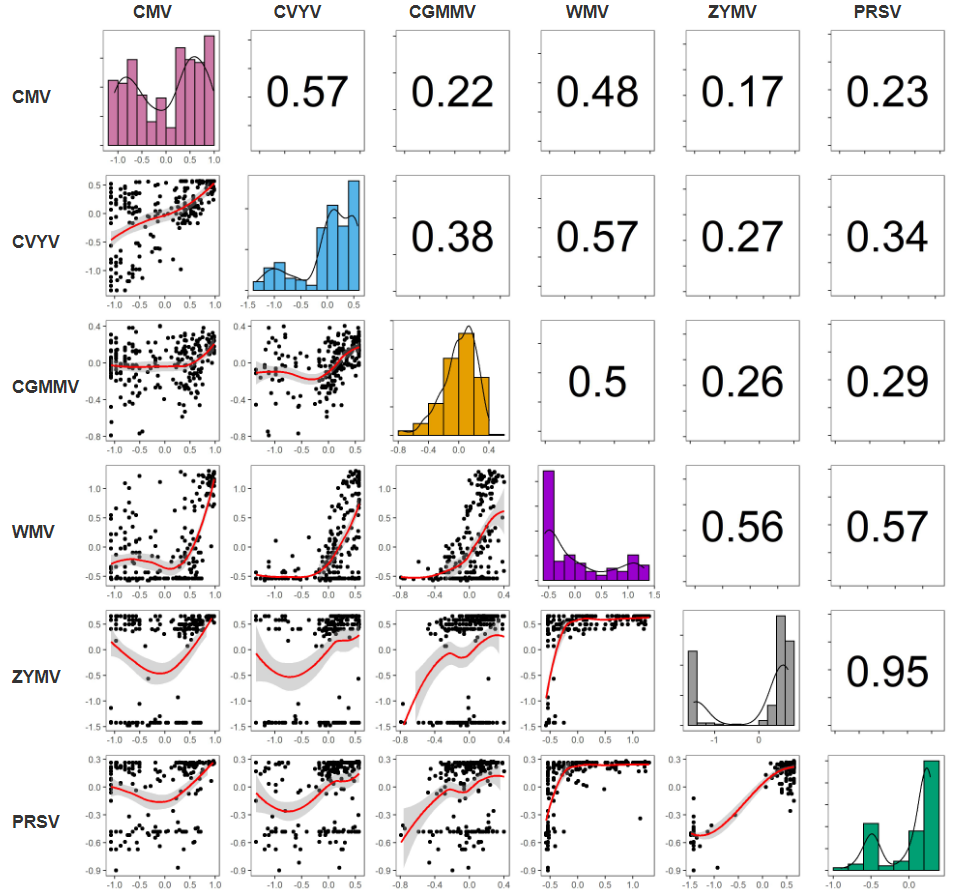

Supplement: Web_Material_uhac184 [file web_material_uhac184.zip › supp_figure_01.png]

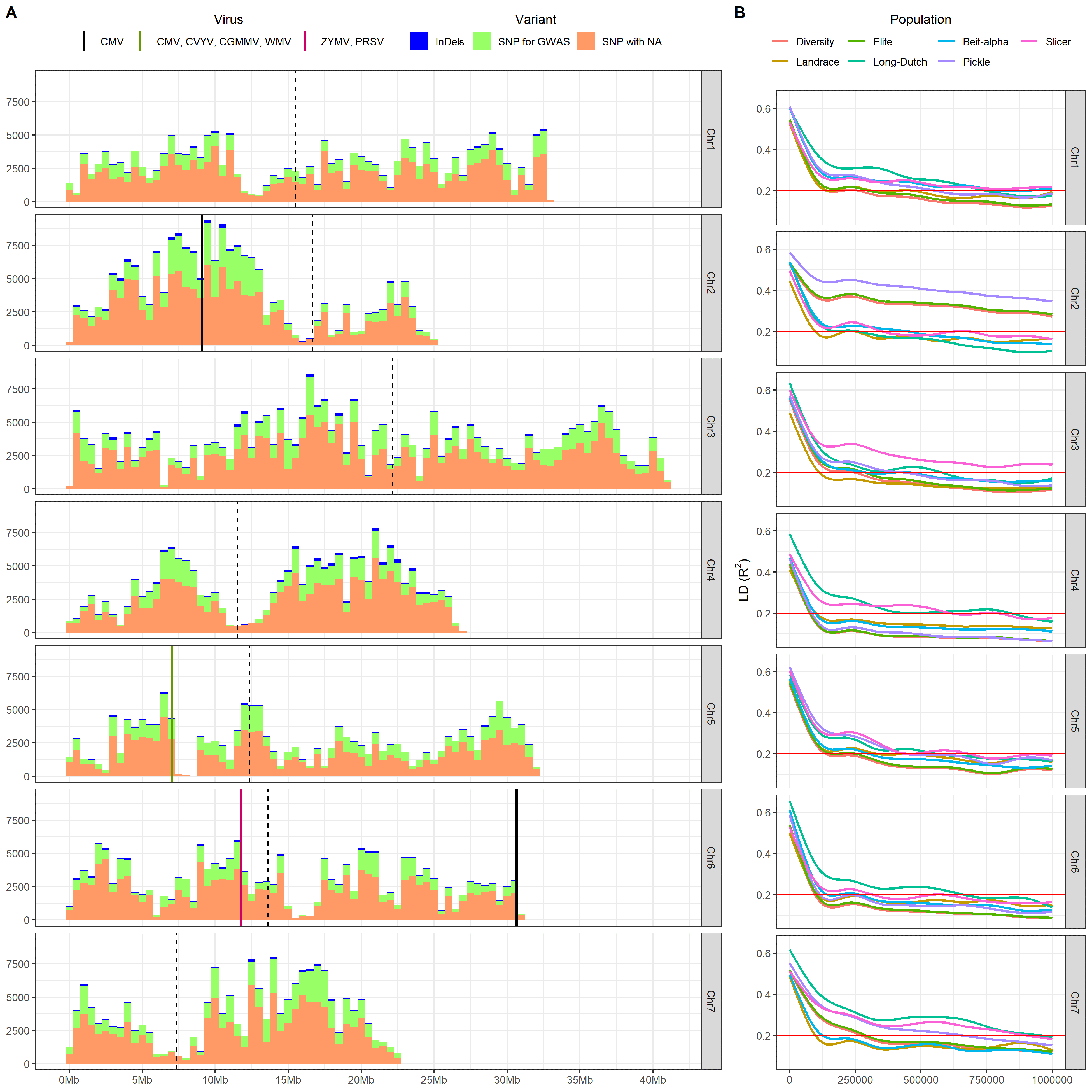

Supplement: Web_Material_uhac184 [file web_material_uhac184.zip › supp_figure_02.png]

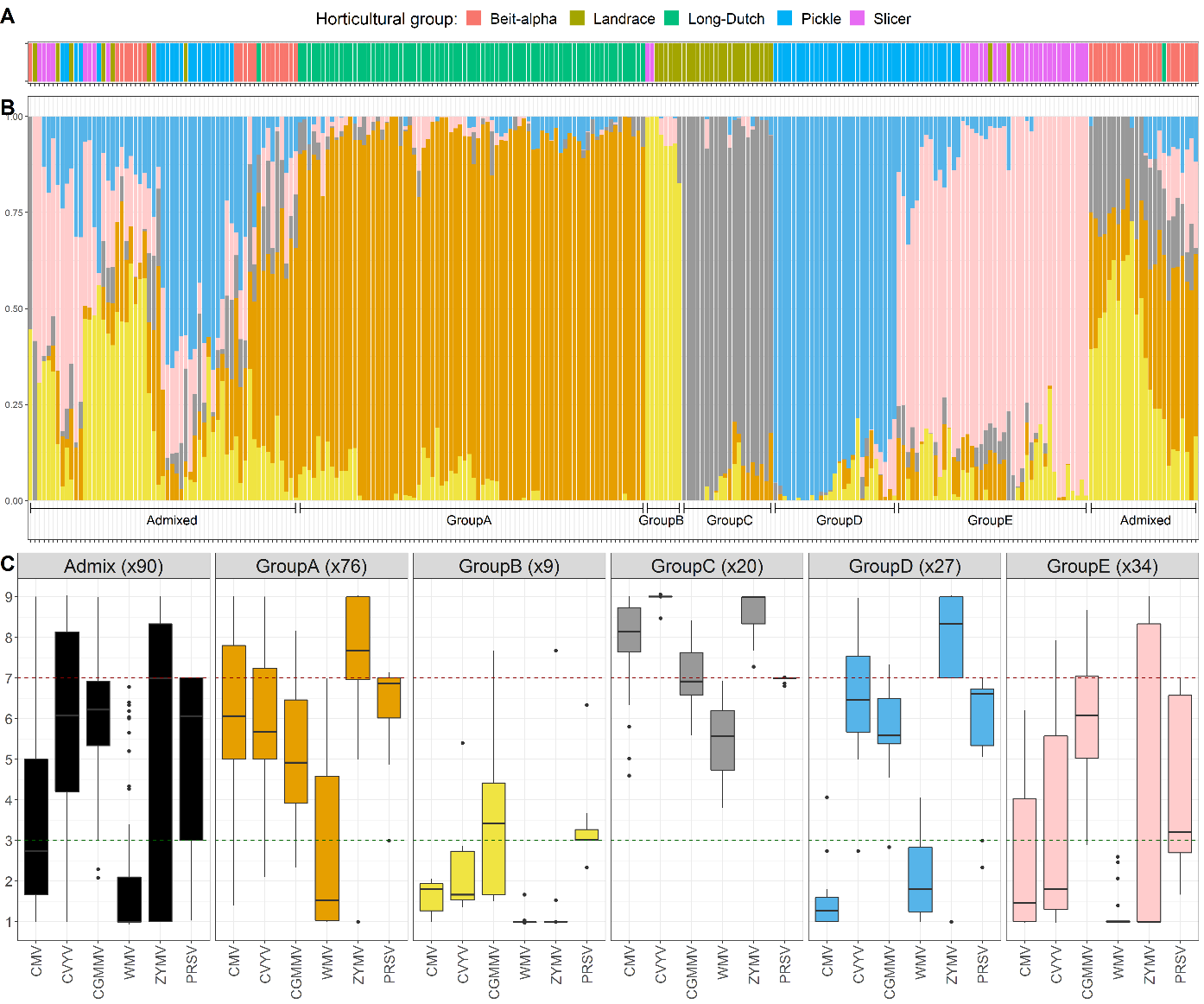

Supplement: Web_Material_uhac184 [file web_material_uhac184.zip › supp_figure_03.png]

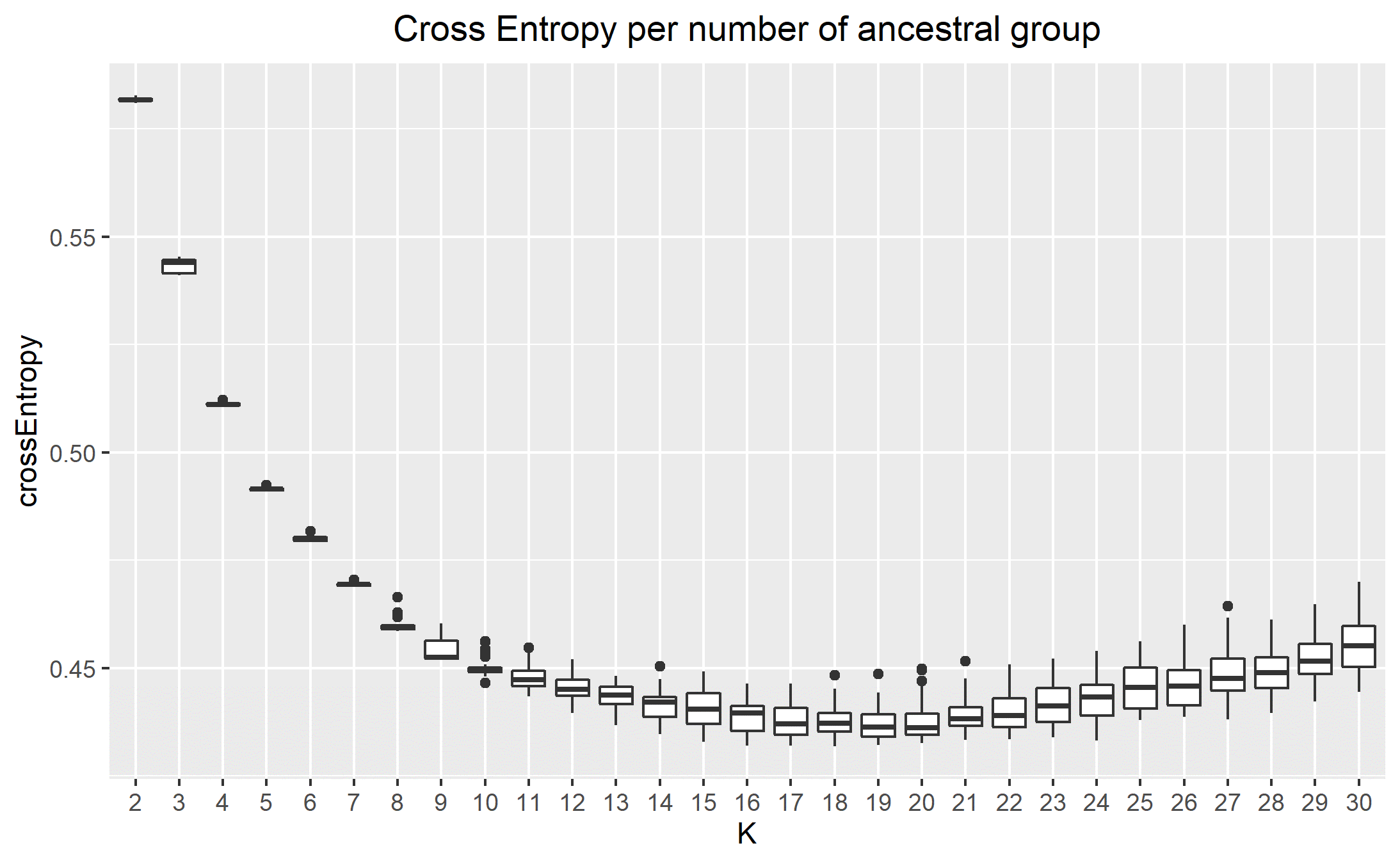

Supplement: Web_Material_uhac184 [file web_material_uhac184.zip › supp_figure_04.png]

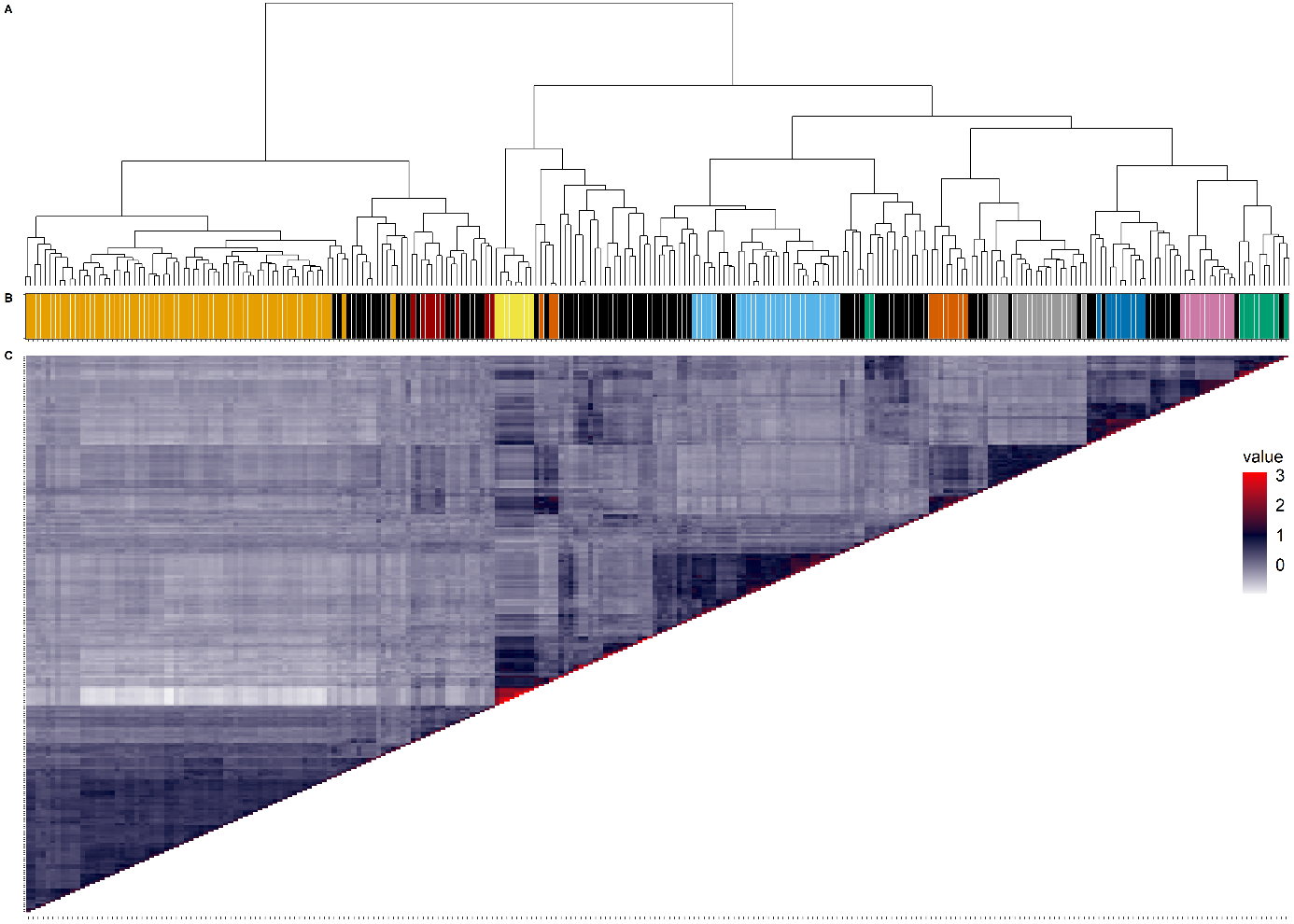

Supplement: Web_Material_uhac184 [file web_material_uhac184.zip › supp_figure_05.png]

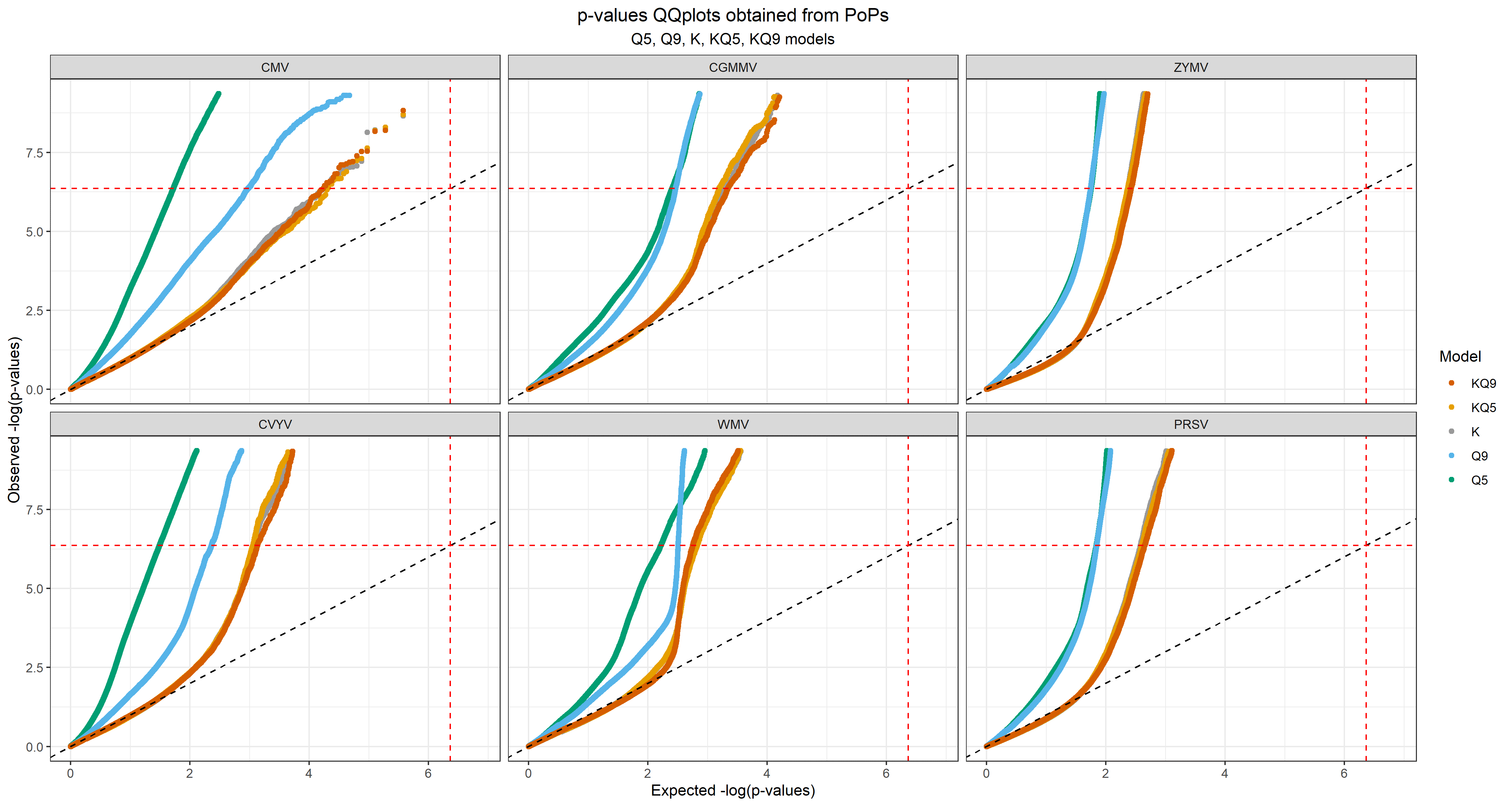

Supplement: Web_Material_uhac184 [file web_material_uhac184.zip › supp_figure_06.png]

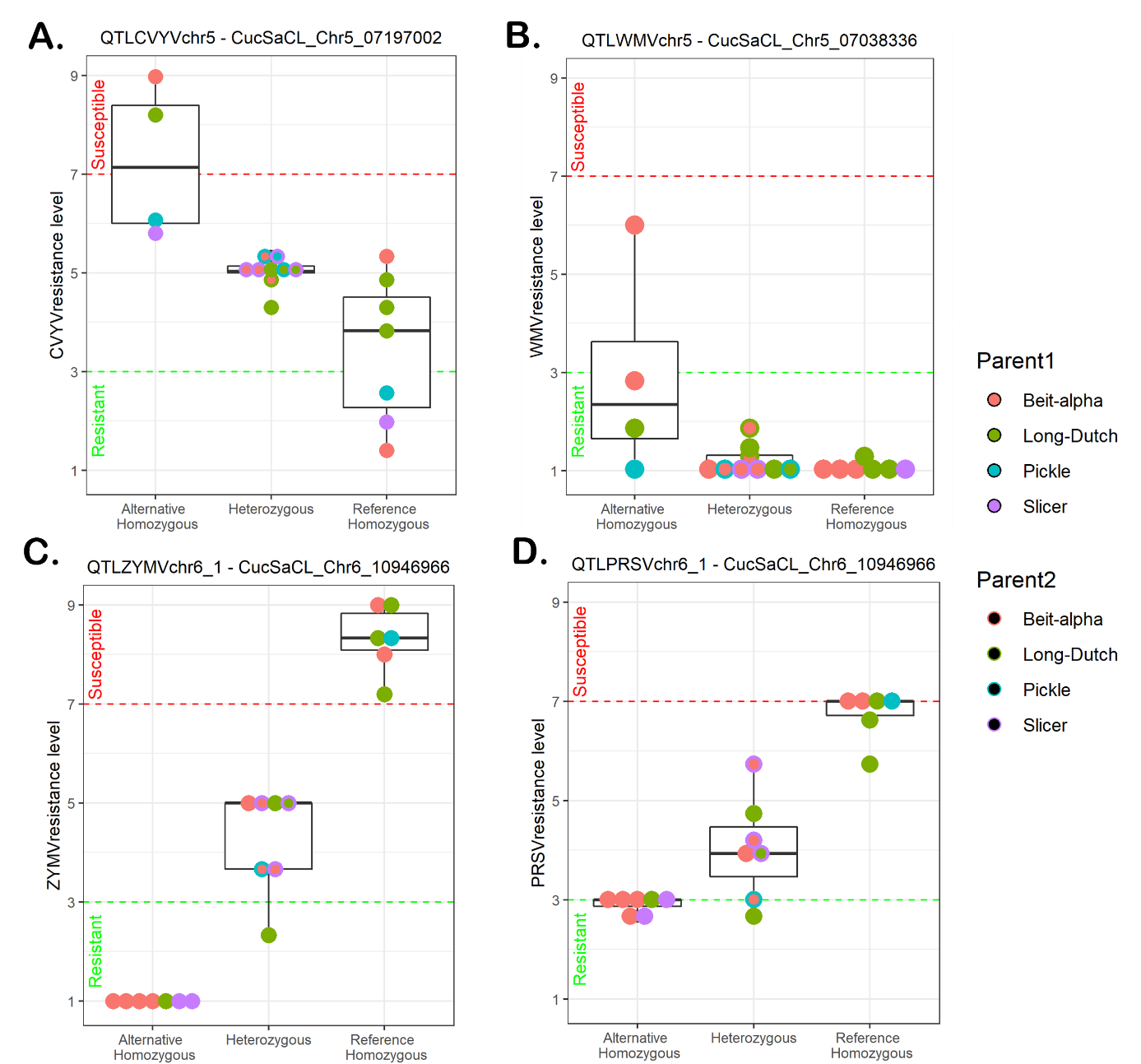

Supplement: Web_Material_uhac184 [file web_material_uhac184.zip › supp_figure_07.png]

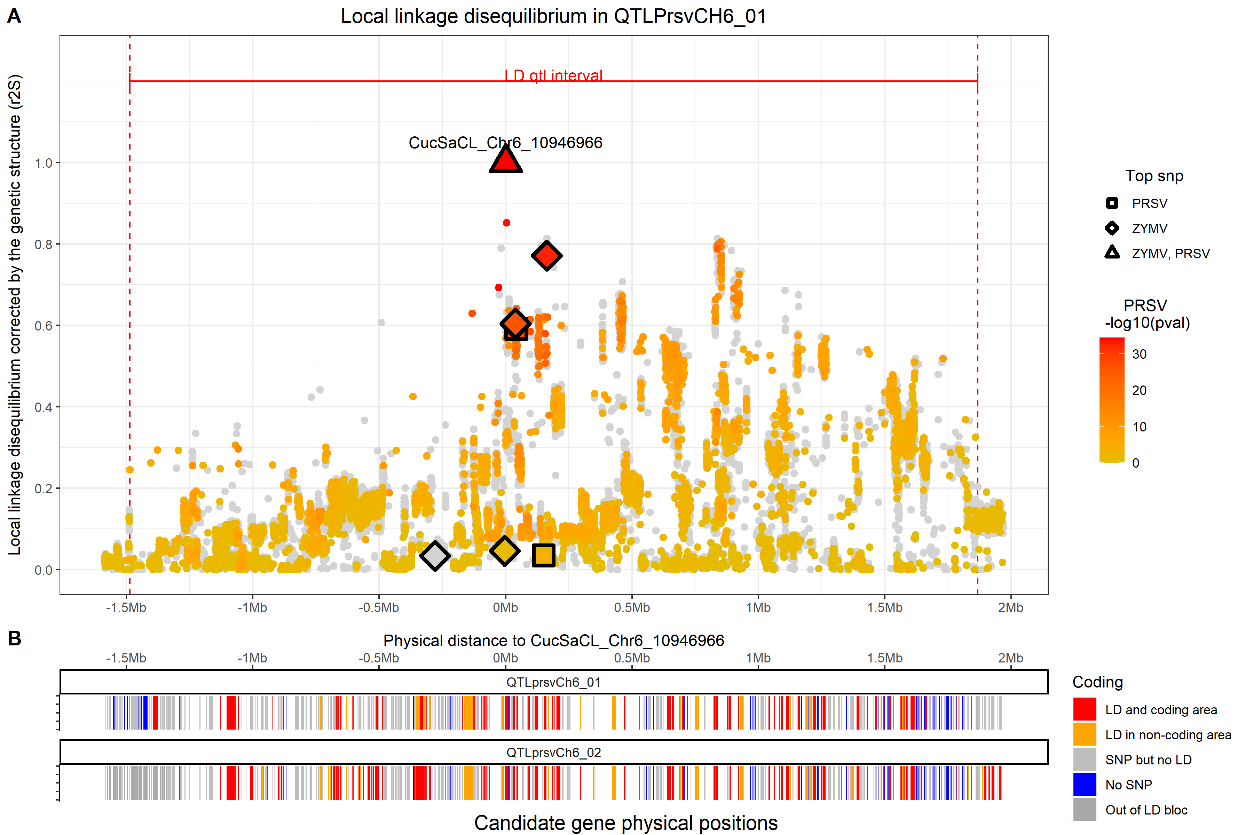

Supplement: Web_Material_uhac184 [file web_material_uhac184.zip › supp_figure_08_I.png]

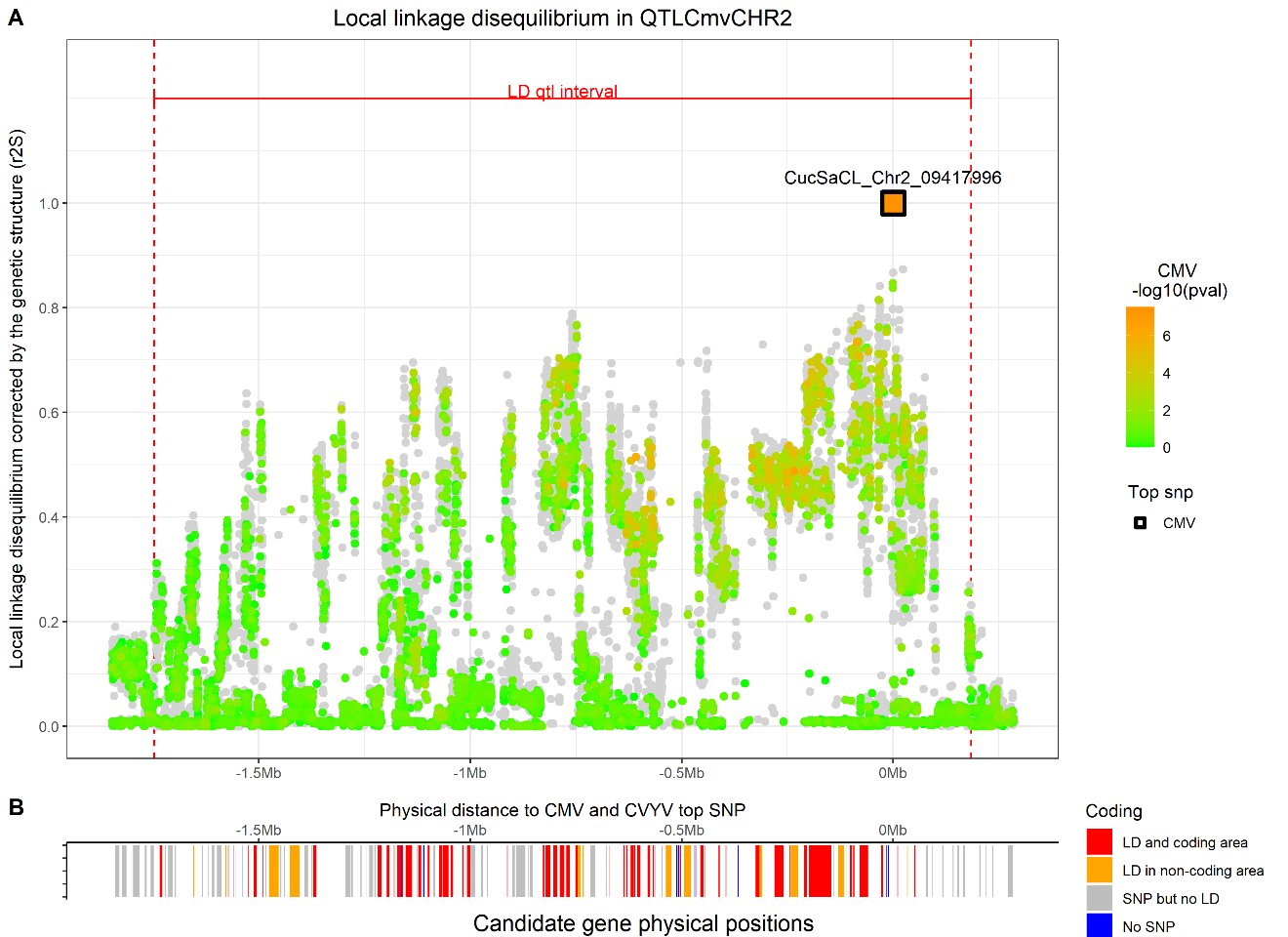

Supplement: Web_Material_uhac184 [file web_material_uhac184.zip › supp_figure_08_II.png]

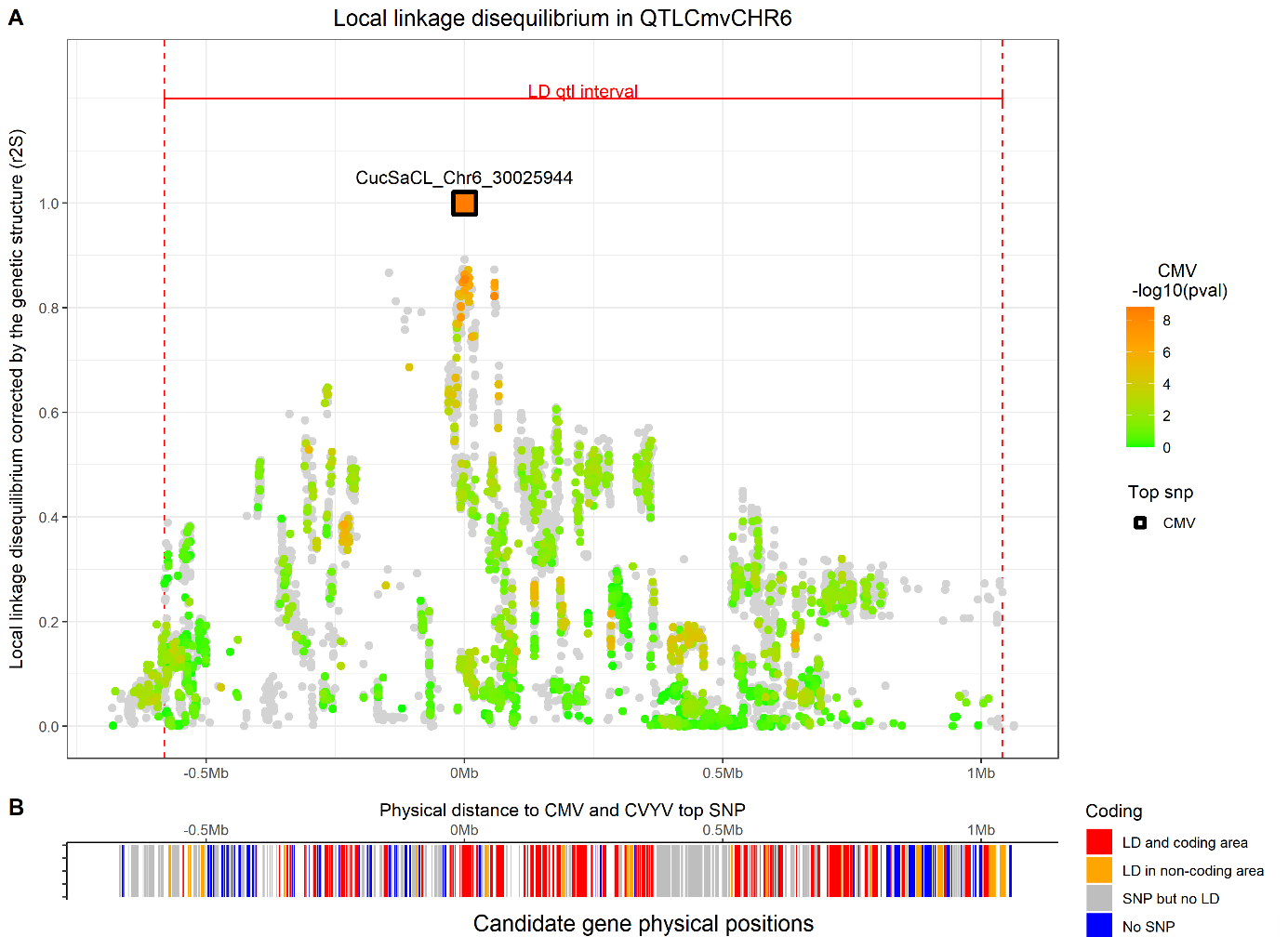

Supplement: Web_Material_uhac184 [file web_material_uhac184.zip › supp_figure_08_III.png]

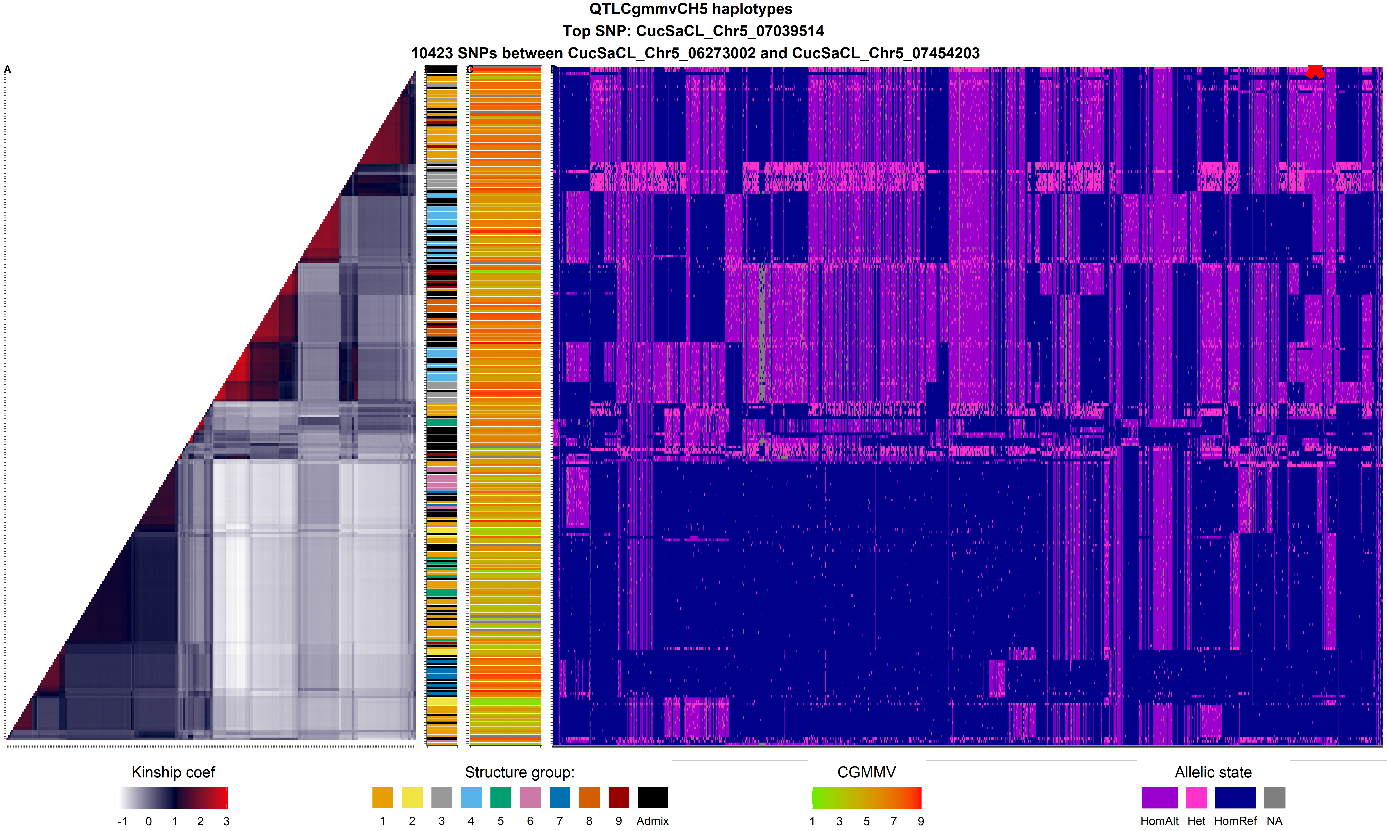

Supplement: Web_Material_uhac184 [file web_material_uhac184.zip › supp_figure_09_I.png]

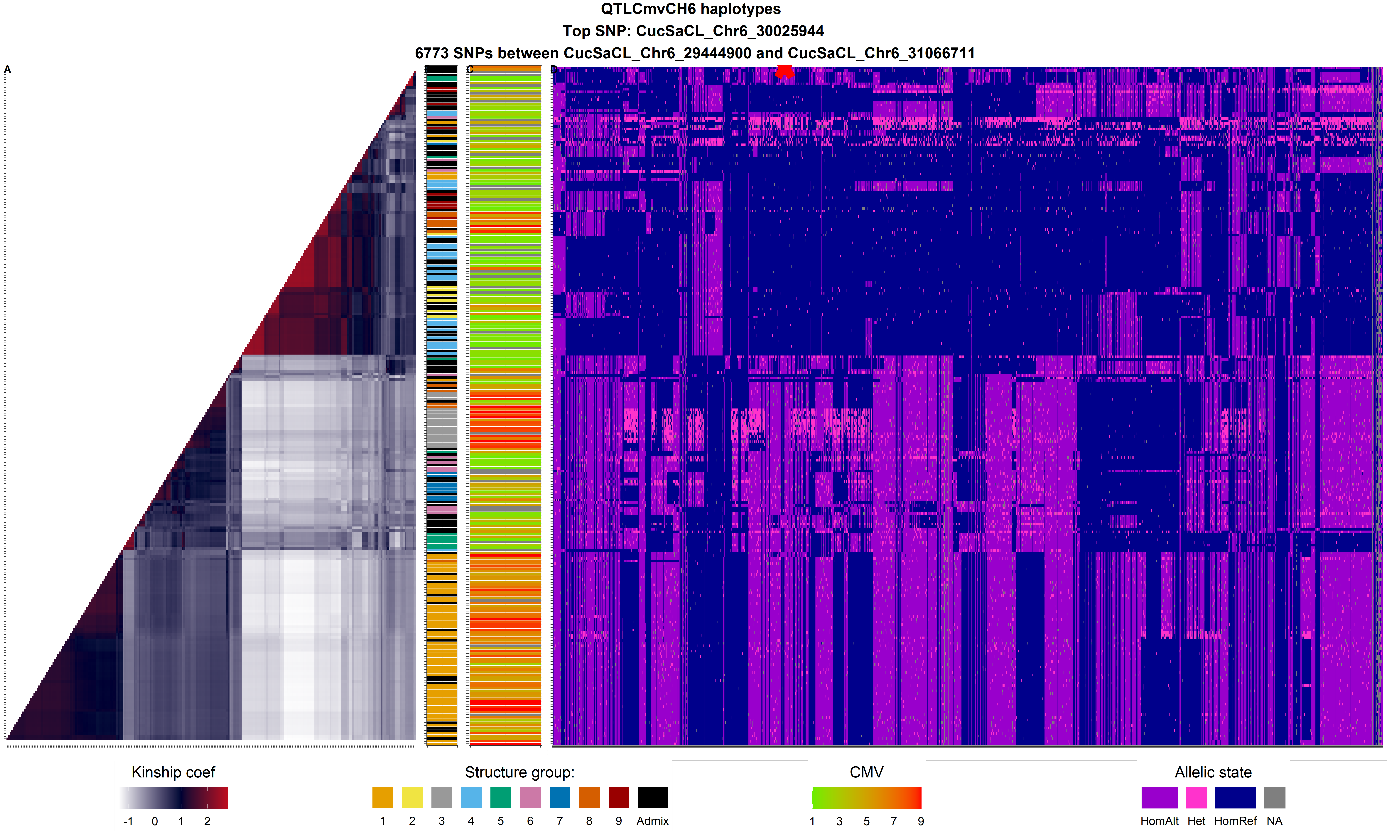

Supplement: Web_Material_uhac184 [file web_material_uhac184.zip › supp_figure_09_II.png]

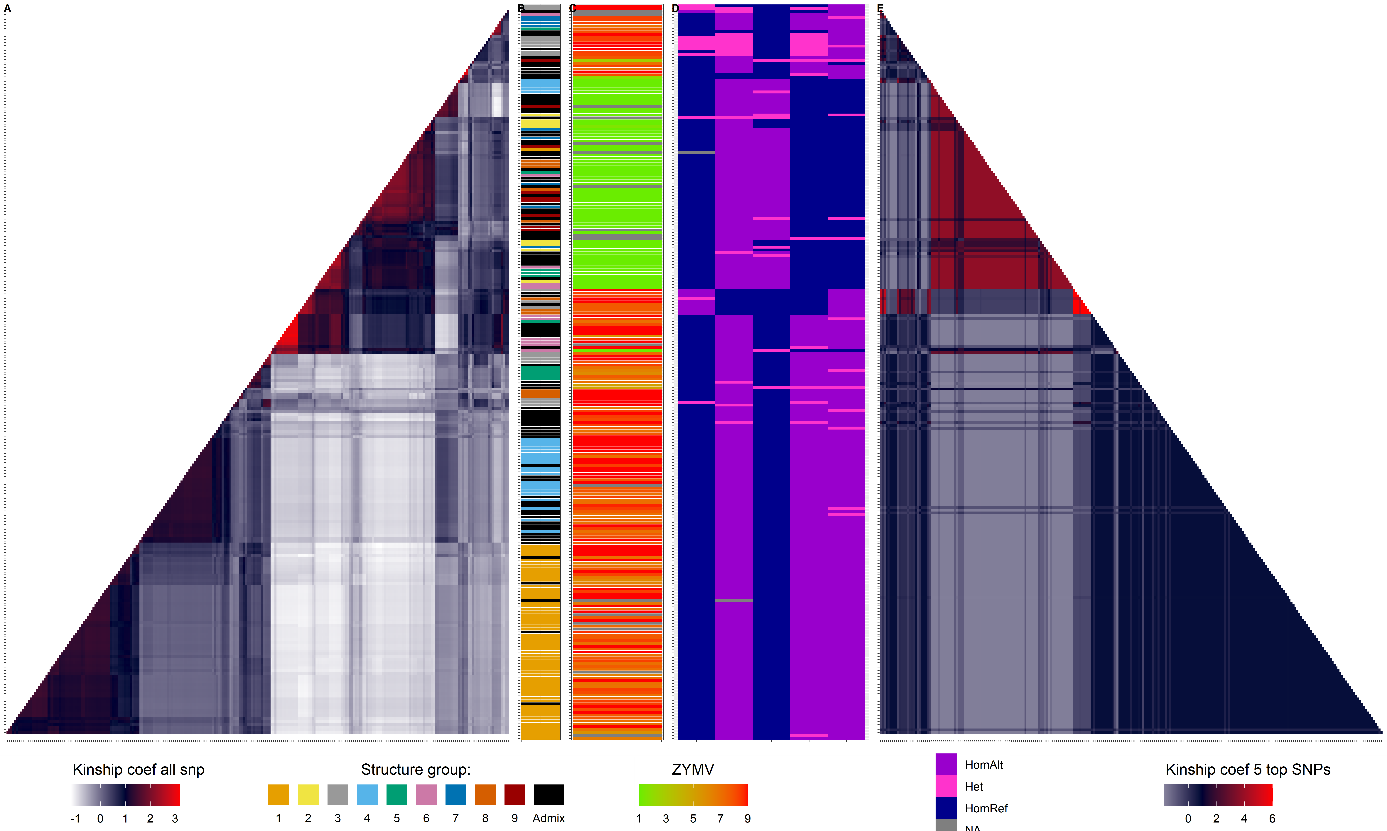

Supplement: Web_Material_uhac184 [file web_material_uhac184.zip › supp_figure_10.png]
